# Supplementary material for: Health insurance utilisation after ischaemic stroke in Sweden: a retrospective cohort study in a system of universal healthcare and social insurance
Source: BMJ Open. 2021 Mar 24;11(3):e043826. doi: 10.1136/bmjopen-2020-043826 (PMC7993163; doi:10.1136/bmjopen-2020-043826)
Supplement: Supplementary data [file bmjopen-2020-043826supp001.pdf]

Table A1. Means and 95% confidence intervals for levels of work absence during the year prior to stroke and the two years following a stroke.

	**Mean**	**95% CI**	
Year prior to ischemic stroke			
<35	42.68409	26.76095	58.60723
35-44	53.65557	44.05273	63.25841
45-54	92.40803	85.53612	99.27993
55-63	125.9772	121.0704	130.8841
First year after ischemic stroke			
<35	153.0585	131.2016	174.9154
35-44	175.7974	163.4365	188.1584
45-54	194.7995	187.5116	202.0874
55-63	211.7774	206.9247	216.6301
Second year after ischemic stroke			
<35	109.7589	86.66959	132.8482
35-44	116.8577	104.0181	129.6973
45-54	156.4038	148.4572	164.3505
55-63	177.6091	172.3538	182.8644
