# Supplementary material for: Health insurance utilisation after ischaemic stroke in Sweden: a retrospective cohort study in a system of universal healthcare and social insurance
Source: BMJ Open. 2021 Mar 24;11(3):e043826. doi: 10.1136/bmjopen-2020-043826 (PMC7993163; doi:10.1136/bmjopen-2020-043826)
Supplement: Supplementary data [file bmjopen-2020-043826supp002.pdf]

Table A2. Sensitivity analysis of multivariable regression assessing association between patient characteristics and net days of absence (sick leave and/or disability pension). Coefficients and 95% CI from regression analysis.

	Coefficient	95% CI		p value
Sex, women	-0,16113	-0,24734	-0,07493	0.000
Age				
<35	[reference]			
35-44	0,188983	-0,10058	0,478549	0,201
45-54	0,250684	-0,01593	0,517297	0,065
55-63	0,175926	-0,08461	0,436462	0,186
ADL dependent	0,12426	-0,19632	0,444836	0,447
Prior stroke (last two years)	0,166235	-0,0371	0,369573	0,109
Inpatient care the year prior to	0,004202			0,015
stroke		0,00082	0,007583	
Living situation				
At home	[reference]			
At home with home				
care	0,157154	-0,09757	0,411879	0,227
At special housing	0,186942	-0,19707	0,570954	0,34
Level of consciousness				
Conscious	[reference]			
Indolent	0,322118	0,122908	0,521328	0,002
Unconscious	0,331179	-0,1402	0,802554	0,169
Single household	0,037734	-0,06697	0,14244	0,48
Disposable income				

Quartile 1	[reference]			
Quartile 2	-0,03551	-0,15224	0,08123	0,551
Quartile 3	0,320181	0,18418	0,456183	0,000
Quartile 4	0,235572	0,125605	0,34554	0,000
Educational level				
≤9 years	[reference]			
10-12 years	-0,04666	-0,14196	0,048639	0,337
≥12 years	-0,23271	-0,34721	-0,1182	0,000
Born outside of the EU	0,161965	0,022217	0,301714	0,023
Marital status				
Married	[reference]			
Never married	0,15266	0,03777	0,26755	0,009
Divorced	0,113267	0,001084	0,225449	0,048
Widowed	-0,15581	-0,40183	0,090218	0,215
