# Supplementary material for: Health insurance utilisation after ischaemic stroke in Sweden: a retrospective cohort study in a system of universal healthcare and social insurance
Source: BMJ Open. 2021 Mar 24;11(3):e043826. doi: 10.1136/bmjopen-2020-043826 (PMC7993163; doi:10.1136/bmjopen-2020-043826)
Supplement: Author's manuscript [file bmjopen-2020-043826.draft_revisions.pdf]

BMJ Open

BMJ Open is committed to open peer review. As part of this commitment we make the peer review history of every article we publish publicly available.

When an article is published we post the peer reviewers' comments and the authors' responses online. We also post the versions of the paper that were used during peer review. These are the versions that the peer review comments apply to.

The versions of the paper that follow are the versions that were submitted during the peer review process. They are not the versions of record or the final published versions. They should not be cited or distributed as the published version of this manuscript.

BMJ Open is an open access journal and the full, final, typeset and author-corrected version of record of the manuscript is available on our site with no access controls, subscription charges or pay-per-view fees (http://bmjopen.bmj.com).

If you have any questions on BMJ Open's open peer review process please email info.bmjopen@bmj.com

BMJ Open

Ischemic stroke and health insurance utilization in a system of universal healthcare and social insurance

Journal:	*BMJ Open*
Manuscript ID	bmjopen-2020-043826
Article Type:	Original research
Date Submitted by the Author:	17-Aug-2020
Complete List of Authors:	Willers, Carl; Karolinska Institute, Department of Neurobiology, Care Sciences and Society<br>Westerlind, Emma; Sahlgrenska Academy, Department of Clinical Neuroscience, Institute of Neuroscience and Physiology<br>Borgström, Fredrik; Karolinska Institute, Department of Learning, Informatics, Management and Ethics, Medical Management Centre<br>von Euler, Mia; Karolinska Institute, Department of Medicine Solna; Karolinska Institute, Department of Clinical Science and Education<br>Sunnerhagen, Katharina; Sahlgrenska Academy, Department of Clinical Neuroscience, Institute of Neuroscience and Physiology
Keywords:	Stroke < NEUROLOGY, HEALTH ECONOMICS, PUBLIC HEALTH

SCHOLARONE™
Manuscripts

I, the Submitting Author has the right to grant and does grant on behalf of all authors of the Work (as defined in the below author licence), an exclusive licence and/or a non-exclusive licence for contributions from authors who are: i) UK Crown employees; ii) where BMJ has agreed a CC-BY licence shall apply, and/or iii) in accordance with the terms applicable for US Federal Government officers or employees acting as part of their official duties; on a worldwide, perpetual, irrevocable, royalty-free basis to BMJ Publishing Group Ltd ("BMJ") its licensees and where the relevant Journal is co-owned by BMJ to the co-owners of the Journal, to publish the Work in this journal and any other BMJ products and to exploit all rights, as set out in our licence.

The Submitting Author accepts and understands that any supply made under these terms is made by BMJ to the Submitting Author unless you are acting as an employee on behalf of your employer or a postgraduate student of an affiliated institution which is paying any applicable article publishing charge ("APC") for Open Access articles. Where the Submitting Author wishes to make the Work available on an Open Access basis (and intends to pay the relevant APC), the terms of reuse of such Open Access shall be governed by a Creative Commons licence – details of these licences and which Creative Commons licence will apply to this Work are set out in our licence referred to above.

Other than as permitted in any relevant BMJ Author's Self Archiving Policies, I confirm this Work has not been accepted for publication elsewhere, is not being considered for publication elsewhere and does not duplicate material already published. I confirm all authors consent to publication of this Work and authorise the granting of this licence.

For peer review only

Ischemic stroke and health insurance utilization in a system of universal healthcare and social insurance

Authors: Carl Willers[1], Emma Westerlind[2], Fredrik Borgström[3], Mia von Euler[4], Katharina Sunnerhagen[2]

1. Department of Neurobiology, Care Sciences and Society, Karolinska Institutet, Stockholm, Sweden

2. Department of Clinical Neuroscience, Institute of Neuroscience and Physiology, Sahlgrenska Academy, University of Gothenburg, Gothenburg, Sweden

3. Department of Learning, Informatics, Management and Ethics, Medical Management Centre, Karolinska Institutet, Stockholm, Sweden **and** Quantify Research, Stockholm, Sweden

4. Clinical Pharmacology Unit, Clinical Epidemiology Division, Department of Medicine Solna, Karolinska Institutet, Stockholm, Sweden **and** Department of Clinical Science and Education, Karolinska Institutet Stroke Research Network at Södersjukhuset, Stockholm, Sweden

Correspondence with Carl Willers, carl.willers@ki.se, +46(0)709 96 43 53

For peer review only

Abstract

Background

Stroke is one of the largest single-condition sources of the global burden of non-communicable disease in terms of disability-adjusted life-years and monetary costs, directly as well as indirectly in terms of informal care and productivity loss. The objective was to assess the population afflicted with ischemic stroke in working age in the context of universal healthcare and social insurance; to estimate the levels of absence from work, the indirect costs related to that, and to assess what patient characteristics that are associated.

Methods

This was a retrospective register-based study; all individuals registered with an ischemic stroke during 2008-2011 in seven Swedish regions, covering the largest cities as well as more rural areas, were included. Individual-level data were used to compute net days of sick leave and disability pension, indirect costs due to productivity loss, and to perform regression analysis on net absence from work to assess the associated factors. Costs related to productivity loss were estimated using the Human Capital approach.

Results

Women had significantly fewer net days than men after multivariate adjustment, and high-income groups had higher levels of sick leave than low-income groups. There were no significant differences for participants regarding educational level, region of birth or civil status.

Conclusion

The burden of stroke in terms of indirect monetary costs amounted to approximately 17.4 kEUR per stroke case first year (12.2 kEUR second year), totaling approximately 169 MEUR in Sweden first year (117 MEUR second year). Income-group differences point to a socioeconomic gradient in the utilization of the Swedish social insurance.

Strengths and limitations of this study

- The present study's underlying data are to a large extent extracted from population registries which implies very high coverage rates and hence a close-to-true picture of the actual population
- Sick-leave costs for stroke patients have been calculated before as well as after the stroke event, which adds important information in terms of incremental costs after stroke
- Regression analysis of net days of absence included clinical as well as socioeconomical variables implying broad adjustments and high possibility to understand the actual drivers
- Informal care is costly, and no estimation of informal care has been included in this study due to lack of reliable data. Furthermore, the actual costs are in fact higher as any cases of sick benefits lasting shorter than 14 days are not registered in the Social Insurance Agency database.
- The Human Capital approach does not take into account that there may be a surplus of unemployed individuals ready to take on the jobs that are left due to disease, and may hence overestimate the productivity loss

Key words: stroke, social insurance, cost analysis, sick leave

For peer review only

Background

Stroke is a leading cause of long-term disability globally and a resource intensive disease, directly and indirectly [1]. Societal costs in terms of productivity loss are immense and have been for the last decade [2]. A non-negligible proportion of stroke patients is still in working age and these incur substantial direct and indirect costs; stroke and ischemic heart disease are the largest sources of the global burden of non-communicable disease in terms of disability-adjusted life-years [1]. Work disability after stroke is generally of more permanent nature compared to other conditions such as ischemic heart disease [3].

The Swedish social insurance covers individuals who live or work in Sweden; all people who are employed and whose employer pay social contributions to the agency are insured against loss of work-related salary up to a standardized ceiling [4]. The social insurance is considered a social security to inhabitants and companies, and thereby also to society [5]. Better understanding of the situation of health insurance utilization and its drivers could help policy makers, governmental bodies and care providers in their work with designing guidelines, monitoring the development of care and optimizing treatment; evidence of the societal impact of stroke incidence, stroke care and recovery is needed to make good decisions on where to invest in the chain-of-care.

The aim with the present study was to map levels of health insurance utilization in relation to ischemic stroke – the year prior to and the two years after – in a health system characterized by universal healthcare and social insurance. Furthermore, the aim was to estimate the distribution of productivity loss associated with stroke across age groups of working age, and to identify key drivers for utilization of health insurance, for individuals afflicted with ischemic stroke (IS).

Methods

Study population and data sources

Seven Swedish regions, covering approximately 65% of the Swedish working age stroke population, contributed with data to the research database. The study population consisted of persons afflicted with ischemic stroke during 2008-2011 and who were aged 18-63 years at the time of stroke. This to only include adults and to allow for two-year follow-up of health insurance utilization, as the general age of retirement in Sweden historically has been 65 years (64,6-64,7 during 2008-2011 [6]). Participants were included independently of degree of sick leave or disability pension prior to stroke.

Stroke cases were identified based on registration of acute ischemic stroke diagnosis (ICD-10 code I63) in regional administrative systems together with a registration in the Swedish Stroke Register (national register for stroke-specific outcomes, reported on optional basis by the caregiver, with a coverage rate of 90% at the time [7]). Follow-up amounted to at least two years for all participants. Relevant data from research registries were linked on individual level, including data on health insurance (Swedish Social Insurance Agency), socioeconomics and mortality (Statistics Sweden).

For peer review only - http://bmjopen.bmj.com/site/about/guidelines.xhtml

Variable definitions

Productivity loss was defined as the sum of sick leave and/or disability pension (early retirement to some degree) in terms of net days (net productivity loss in terms of full working days; sick leave can formally be granted to an extent of 25%, 50%, 75% or 100% per day). Absence was calculated based on available information from the Swedish Social Insurance Agency; sick leave lasting 14 days or shorter is financed by the employer, and not available in the national registry. Hence only sick leave periods lasting longer than 14 days were included in the analysis.

Information on prior stroke, history of atrial fibrillation and hypertension was based on administrative data, including diagnosis registration, for two years prior to the stroke. Information on dependency for activities of daily living (ADL) was based on data from the Swedish Stroke Register (in need of help with either dressing or toilet visits rendered an ADL value of 1, else 0), as was the computation of the modified Rankin Scale (mRS) which was made in accordance with a published and validated method for approximation [8]. Due to low coverage rate of the National Institutes of Health Stroke Severity (NIHSS) in the register (<50% complete for this study population), an approximation of stroke severity was captured via information on level of consciousness at hospital arrival (a version of the Reaction Level Scale which is used in the Swedish Stroke Register) which has been shown to be a good approximation of NIHSS for prediction of e.g. mortality [9].

Indirect costs were calculated based on the Human Capital approach [10]; it was assumed that indirect costs after stroke for the individual participant was equivalent to the sum of income the participant would receive if not having a stroke (i.e. based on number of net days of absence and the level of income per day during the year prior to stroke), and the employer contribution that the employer pays for their employees (31.42% of the salary [11]). Indirect costs related to the first 14 days of the included cases were also estimated and included in the analysis. The authors did however not have any information whether the individual participant was in fact employed at the time of stroke. Information on disposable income was available on individual level and was used as explanatory variable in the regression analysis, then categorized into quartiles. The exchange rate for Swedish krona (SEK) per Euro (EUR) was set to 0.11198, which was the official exchange rate on December 31st, 2011 [12].

Statistical analysis

Estimation of indirect costs related to productivity loss was made per age group (age spans set prior to the analysis; <35, 35-44, 45-54, 55-63 years of age at time of stroke). The estimate was based on individual-level income data for the study population. Participants deceased during follow-up were excluded from the analysis.

In addition, multivariate regression analysis was performed, adjusting for a broad range of select factors of relevance (presented in Table 2). Health insurance utilization was analyzed in terms of sick leave and disability pension (early retirement due to disability) as count variables with negative binomial regression. The reason for treating it as a count variable was that the decision on the total amount of sick leave is iterative and based on a joint agreement between the doctor and the patient done during several occasions.

For peer review only

Results

The study population amounted to 6 637 individuals, whereof 35% women. The study population was split into four age groups (Table 1). The elder the age group, the larger the volume of participants per age group; the youngest age group, <35 years, consisted of 180 individuals, and the eldest age group consisted of 4 174 individuals. The younger the age group, the larger the proportion of women; 52% in the youngest age group, and 33% in the eldest age group. The prevalence of atrial fibrillation and hypertension seemed to increase with higher age, according to registration in medical records. The eldest age group (55-63 years at the time of stroke) versus the youngest (<35 years at stroke) showed a prevalence of 10.9% versus 1.1% (atrial fibrillation) and 54.1% versus 3.9% (hypertension) respectively. Individual income (quartiles 1, 2 and 3) was highest in the group of participants aged 35-44 at the time of stroke. The youngest age group was the most educated in terms of years spent in school with 40,7% having studied for 12 years or more.

For peer review only

Table 1. Baseline characteristics of the study population, per age group

	Age groups			
	**<35 yrs**	**35-44 yrs**	**45-54 yrs**	**55-63 yrs**
Sex, proportion women (%)	52	42	37	33
ADL dependent (%)	1.7	1.1	1.5	3.0
Prior stroke, last two years (%)	3.4	4.0	4.3	4.6
Inpatient care the year prior to stroke (days)	3.25	2.78	3.92	3.81
Level of consciousness (%)				
Conscious	89.8	94.0	93.4	93.9
Indolent	5.7	5.2	5.2	4.9
Unconscious	4.5	0.8	1.4	1.2
Atrial fibrillation, prior to stroke (%)	1.1	2.9	5.4	10.9
Hypertension, prior to stroke (%)	3.9	23.0	41.1	54.1
Reperfusion treatment (thrombolysis or thrombectomy, %)	15.3	14.3	13.0	9.9
Single household (%)	27.3	21.7	30.8	34.4
Disposable income (EUR, nominal amount)				
Q1	12 788	16 786	14 401	14 199
Q2	17 217	24 468	21 646	20 935
Q3	25 330	32 256	29 787	29 182
Educational level (%)				
≤9 years	16.95	13.79	27.37	31.04
10-12 years	42.37	53.31	50	46.41
≥12 years	40.68	32.9	22.63	22.55

For peer review only

Born outside the EU (%)	10.0	15.0	15.6	8.7
Sick leave the year prior to stroke (net days)	17.7	19.1	18.4	17.3
Disability pension the year prior to stroke (net days)	25.0	34.5	74.0	108.7
Observations (n)	180<br>(3%)	548<br>(8%)	1 735<br>(26%)	4 174<br>(63%)

For peer review only

The individual levels of absence from work due to sickness the year prior to stroke varied between the age groups (Figure 1). The youngest age groups showed average absence of 42.7 and 53.6 net days respectively, whilst individuals in ages 45-54 at stroke presented with 92.4 net days of absence the year prior to stroke. Individuals aged 55-63 at stroke had 126.0 net days of absence the year prior to stroke. The higher the age group, the higher the ratio of disability pension; the levels of net days prior to stroke were made up of 59% disability pension (<35 years), 64% disability pension (35-44 years), 80% disability pension (45-54 years) and 86% disability pension (55-63 years) respectively (the remaining proportion of net days regarded sickness benefit).

During the first year after stroke, levels of absence increased significantly from the year before, but overall, levels were on more similar levels across the different age groups; 153.1 (<35 years at stroke), 175.8 (35-44 years at stroke), 194.8 (45-54 years at stroke), and 211.8 (55-63 years at stroke) net days respectively. The increase between the year prior to stroke versus after stroke was highest in the youngest age group (3.6 times higher) and lowest in the eldest age group (1.7 times higher).

Levels of absence the second year after stroke were significantly lower than levels the first year after stroke. The largest relative drop in absence was found in the second youngest age group (35-44 years) and amounted to -33.5%. The age group with the lowest relative increase in the second year compared to the year prior to stroke was the eldest age group with 41% more net days.

Figure 1. Average health insurance levels utilized per individual, before and after stroke per age group: net days of sick leave and/or disability pension.

For peer review only

The loss of productivity, calculated based on individual participants' income together with additional employer contributions, amounted to 110 Million Euros during the first year after stroke (Figure 2). The indirect costs were 27% lower during the second year after stroke and amounted to 76 Million Euros. The age-group share of total was similar during the first and second year although the second youngest age group was relatively less costly during the second year after stroke.

The average indirect cost per participant per year during the first and second year amounted to 11.6 kEUR and 7.3 kEUR for the youngest age group, 16.6 kEUR and 9.0 kEUR for participants aged 35-44, 16.8 kEUR and 11.5 kEUR for participants aged 45-54, and 18.0 kEUR and 13.2 kEUR for participants aged 55-63 at the time of stroke.

Figure 2. Indirect costs in terms of productivity loss per age group years 1-2 after stroke, based on individual income data from Statistics Sweden. Proportion based on population contribution reflects whether the age-group share of total costs is representative or not (age group under-represented if <100% and over-represented if >100%).

When adjusting for other relevant factors of potential association with levels of absence after stroke, belonging to a certain age group was not significantly associated to differences in absence from work due to sick leave or disability pension.

Women had significantly fewer net days of absence during the year after stroke compared to men, after multivariate adjustments. Participants in the lowest (first) income quartile had significantly lower absence levels compared to participants in the second, third and fourth quartile. There were no significant differences for participants regarding educational level, region of birth or civil status.

For peer review only

Table 2. Drivers of post-stroke health insurance utilization (coefficients and 95% CI from regression analysis): net days of absence (sick leave and/or disability pension)

	Coefficient	95% CI		p value
Sex, women	-0.085	-0.167	-0.004	0.040
Age				
<35	[reference]			
35-44	0.144	-0.114	0.402	0.274
45-54	0.158	-0.081	0.397	0.196
55-63	0.150	-0.085	0.386	0.211
ADL dependent	0.020	-0.299	0.340	0.902
Prior stroke (last two years)	-0.059	-0.257	0.140	0.561
Inpatient care the year prior to stroke	0.001	-0.002	0.004	0.562
Net days of absence the year prior to stroke (sick leave and/or disability pension)	0.003	0.003	0.003	0.000
Living situation				
At home	[reference]			
At home with home care	-0.067	-0.321	0.188	0.607
At special housing	-0.067	-0.436	0.303	0.724
Stroke severity				
Conscious	[reference]			
Indolent	0.398	0.211	0.586	0.000
Unconscious	0.353	-0.083	0.790	0.113
Single household	0.013	-0.084	0.109	0.794

Disposable income				
Quartile 1	[reference]			
Quartile 2	0.309	0.201	0.417	0.000
Quartile 3	0.417	0.302	0.532	0.000
Quartile 4	0.218	0.097	0.340	0.000
Educational level				
≤9 years	[reference]			
10-12 years	0.022	-0.068	0.112	0.632
≥12 years	-0.079	-0.190	0.031	0.161
Born outside of the EU	0.033	-0.092	0.157	0.609
Marital status				
Married	[reference]			
Never married	0.076	-0.028	0.180	0.153
Divorced	0.030	-0.075	0.134	0.575
Widowed	-0.106	-0.366	0.154	0.425

For peer review only

Discussion

The burden associated with stroke is immense – in terms of Health-Related Quality of Life [1] as well as money [13-15]. In addition to severe personal suffering, stroke affects society by putting a significant burden on direct healthcare resources but also by indirect, major and long-lasting productivity loss. According to the present study, first-year indirect costs in terms of productivity loss and employer contributions amount to on average 15 400 Euros per stroke case in younger working ages (up to 44 years) and to 17 600 Euros per stroke case in the older working ages (45-63 years at stroke). Second-year indirect monetary costs amounted to on average 8 600 Euros per stroke case in the younger group and to 12 700 Euros per stroke case in the older group. The first-year indirect monetary costs for stroke in the present study totaled 110 Million Euros. Assuming the equivalent incidence, and equivalent levels of income for the rest of Sweden renders an estimate of approximately 169 Million Euros annually – including only the first year after stroke. The costs are not limited to the first or second year (indirect monetary costs of 76 MEUR for the study population, estimated to 117 MEUR for the full Swedish population), but expected to last several years after the stroke event [16]. Indirect costs related to stroke incidence in Sweden have been assessed in previous studies and found to be a minor proportion of the total cost [13-15]. These studies did however include persons of all ages afflicted with stroke, and likely the indirect costs are significantly higher for the group in general working age as included in the present study.

Levels of sick leave the year prior to stroke were similar between the age groups (17.3-19.1 net days), whilst absence due to disability pension the year prior to stroke varied significantly (25.0-108.7 net days); the older the age group, the higher absolute and relative levels of disability pension. The levels of net absence during the year prior to stroke points at the fact that participants active on the labor market were more afflicted with disease than the average general population; pre-stroke levels of sick leave (absence excluding disability pension) in the study population amounted to 17.7 net days (average of all age groups) whilst the national average for the same time period amounted to 6.0 net days (2010) [17]. The patterns are the same looking at the health insurance as a whole; total absence in the study population the year prior to stroke amounted to more than three times the total absence in the general population for the study population (109.0 net days compared to 29.5 net days 2010 [17]). The proportion of women over the different age groups differed; the younger the age group, the higher the proportion of women. Higher proportion of women in younger age categories may be related to impacted hormone levels from contraceptives or pregnancy, e.g. changed balance in coagulation factors or hypertensive crisis [18].

Sex and stroke severity were significantly associated with the level of absence the year after stroke; women were less absent on sick leave and/or disability pension as were participants who were conscious at hospital arrival compared to those who were indolent. The finding that female sex was significantly associated with lower levels of absence the year after stroke stood out compared to the general population; according to the Social Insurance Agency, women have higher risk for being on sick leave, a difference which has continued to increase since 2010 [19]. Sick leave figures in public reports are often presented without multivariate adjustments which was the case in the present study. Furthermore, disposable income showed strong association with absence after stroke; participants in the lowest income group had significantly lower levels of absence compared to all other income groups.

Absence the year prior to stroke was positively correlated with absence the year after stroke. Interestingly, when this baseline characteristic was not taken into account, several socioeconomic indicators showed significant association with absence the year after stroke, including educational level, birth region and civil status.

For peer review only - http://bmjopen.bmj.com/site/about/guidelines.xhtml

The actual costs are in fact higher as any cases of sick benefits lasting shorter than 14 days are not registered in the Social Insurance Agency database. Furthermore, informal care is costly, and no estimation of informal care has been included in this study due to lack of reliable data. During 2008, significant changes were implemented in the Swedish health insurance to limit the length of time possible to receive support for loss of income due to disability. These changes were implemented at the beginning of the time period assessed in the present study and could be expected to influence the estimation of costs equivalently over the time period studied [20].

The fact that low-income individuals had fewer net days of sick leave is important to note as this points to a socioeconomic gradient in the utilization of the general health insurance. There are several different factors that may impact and drive that gradient, but it is likely that factors such as higher general dependence on income in lower-income professions and higher general knowledge regarding the social insurance system in higher-income groups are of relevance to explain this gradient [21].

Conclusions

Individuals that are afflicted with stroke in working age have a significantly higher pre-stroke level of health insurance (sick leave or disability pension) utilization than the average in Sweden. The monetary burden of stroke is very high; the first-year indirect costs for an individual in working age amounts to more than one third of the GDP per capita (reference year 2011). There is possibly a socioeconomic gradient in the utilization of Swedish health insurance among stroke patients; high-income individuals generally had higher levels of sick leave than did low-income individuals, while simultaneously adjusting for other relevant factors on patient level. Continuous assessments of the social insurance utilization among stroke patients – as well as for other conditions – by governmental institutions are encouraged, to follow up on the equity in its design and practical implications.

For peer review only

List of abbreviations

ADL – activities of daily living

EU – European Union

GDP – Gross Domestic Product

IS – ischemic stroke

EUR - Euros

Contributorship

CW and KSS conceived the idea to the study. CW performed the analyses and drafted the manuscript. All authors contributed to the final version and approved the submitted version.

Competing interests

None declared.

Funding

This study was supported by grants from the Swedish Research Council (VR2017-00946), the Swedish Heart and Lung Foundation, the Swedish Brain Foundation, the Promobilia Foundation, the Norrbacka-Eugenia Foundation as well as by grants from the Swedish state under an agreement between the Swedish government and the county councils, the ALF agreement (ALFGBG-718711).

Data sharing

The dataset generated and analysed during the current study is not publicly available due to Swedish law; access to the dataset was given to the researchers under the condition of timely valid ethical approval. Additional information regarding the availability is available from the corresponding author upon request.

Acknowledgements

We are grateful to the Swedish Stroke Register (www.riksstroke.org) and the different healthcare regions for providing data for this study. We also wish to thank all patients, caregivers, reporting units, and coordinators in Riksstroke as well as Riksstroke steering committee.

Patient and Public Invlovement

It was not appropriate or possible to involve patients or the public in the design, or conduct, or reporting, or dissemination plans of our research.

For peer review only

References

1. *Global, regional, and national burden of stroke, 1990-2016: a systematic analysis for the Global Burden of Disease Study 2016.* Lancet Neurol, 2019. **18**(5): p. 439-458 GBD Stroke Collaborators.
2. Luengo-Fernandez, R., et al., *Economic burden of stroke across Europe: A population-based cost analysis.* European Stroke Journal, 2019: p. 2396987319883160.
3. Ervasti, J., et al., *Permanent work disability before and after ischaemic heart disease or stroke event: a nationwide population-based cohort study in Sweden.* BMJ Open, 2017. **7**(9): p. e017910.
4. *The Swedish social insurance during 2018 (Socialförsäkringen i siffror 2018)*. 2018, The Swedish Social Insurance Agency: Stockholm.
5. *About the Swedish social insurance*, T.S.S.I. Agency, Editor. 2019.
6. *Average age of retirement and exit from the Swedish labor market (Medelpensioneringsålder och utträdesålder)*. 2016, The Swedish Pensions Agency.
7. Söderholm, A., et al., *Validation of Hospital Performance Measures of Acute Stroke Care Quality. Riksstroke, the Swedish Stroke Register.* Neuroepidemiology, 2016. **46**(4): p. 229-34.
8. Eriksson, M., et al., *Assessment of functional outcome in a national quality register for acute stroke: can simple self-reported items be transformed into the modified Rankin Scale?* Stroke, 2007. **38**(4): p. 1384-6.
9. Bray, B.D., et al., *Derivation and external validation of a case mix model for the standardized reporting of 30-day stroke mortality rates.* Stroke, 2014. **45**(11): p. 3374-80.
10. Drummond, M.F., *Methods for the economic evaluation of health care programmes*. 3rd edition ed. 2005, Oxford: Oxford University Press.
11. *Size of employer contributions*. [cited 2019 25 October]; Description of employer contribution for persons in general working age and its components:[Available from: https://www4.skatteverket.se/rattsligvagledning/edition/2014.14/1333.html.
12. *Oanda Currency Converter*. 2019 [cited 2019 November 14]; Available from: https://www1.oanda.com/currency/converter/.
13. Lekander, I., et al., *Relationship between functional disability and costs one and two years post stroke.* PLoS One, 2017. **12**(4): p. e0174861.
14. Ghatnekar, O., et al., *Cost of stroke in Sweden: an incidence estimate.* Int J Technol Assess Health Care, 2004. **20**(3): p. 375-80.
15. Ghatnekar, O., et al., *Costs for stroke in Sweden 2009 and developments since 1997.* Int J Technol Assess Health Care, 2014. **30**(2): p. 203-9.
16. Gloede, T.D., et al., *Long-term costs of stroke using 10-year longitudinal data from the North East Melbourne Stroke Incidence Study.* Stroke, 2014. **45**(11): p. 3389-94.
17. *Social Insurance Report 2011:6*. 2011, The Swedish Social Insurance Agency.
18. Cordonnier, C., et al., *Stroke in women - from evidence to inequalities.* Nat Rev Neurol, 2017. **13**(9): p. 521-532.
19. *Social Insurance Report 2018:2*. 2018, The Swedish Social Insurance Agency.
20. *Follow-up report of the health insurance reform, reference number 037795-2011 (Försäkringskassans uppföljning av sjukförsäkringsreformen – delredovisning)*. 2011, The Swedish Social Insurance Agency.
21. *Insured but not reimbursed - a report on the under-utilization of the union-based health insurance (Försäkrad men utan ersättning - en granskning av underutnyttjandet av den kollektivavtalade sjukförsäkringen); 2018:7*. 2018, The Swedish Social Insurance Inspectorate.

For peer review only

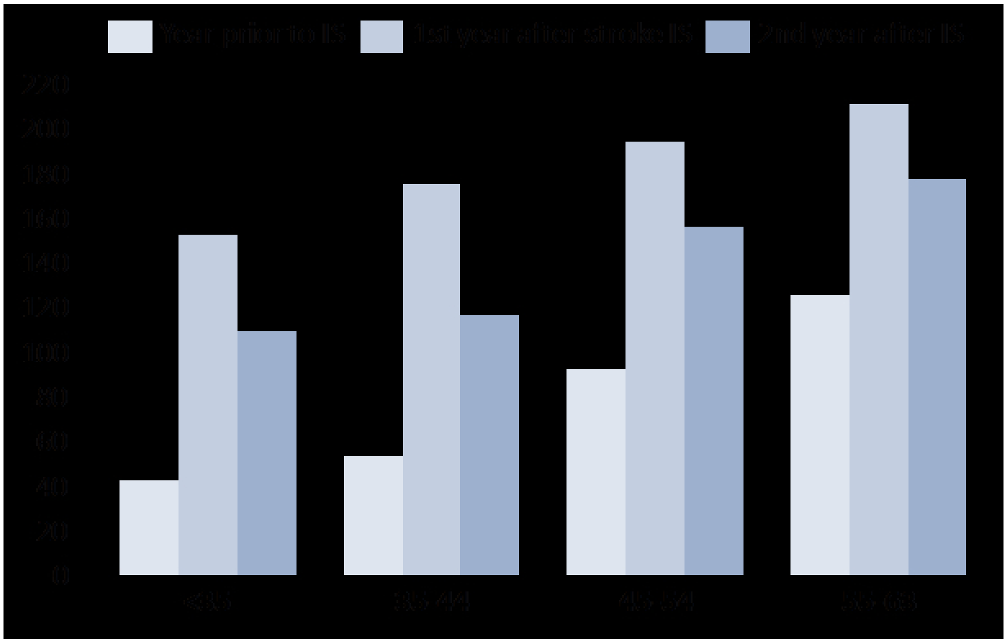

Figure 1. Average health insurance levels utilized per individual, before and after stroke per age group: net days of sick leave and/or disability pension.

161x102mm (96 x 96 DPI)

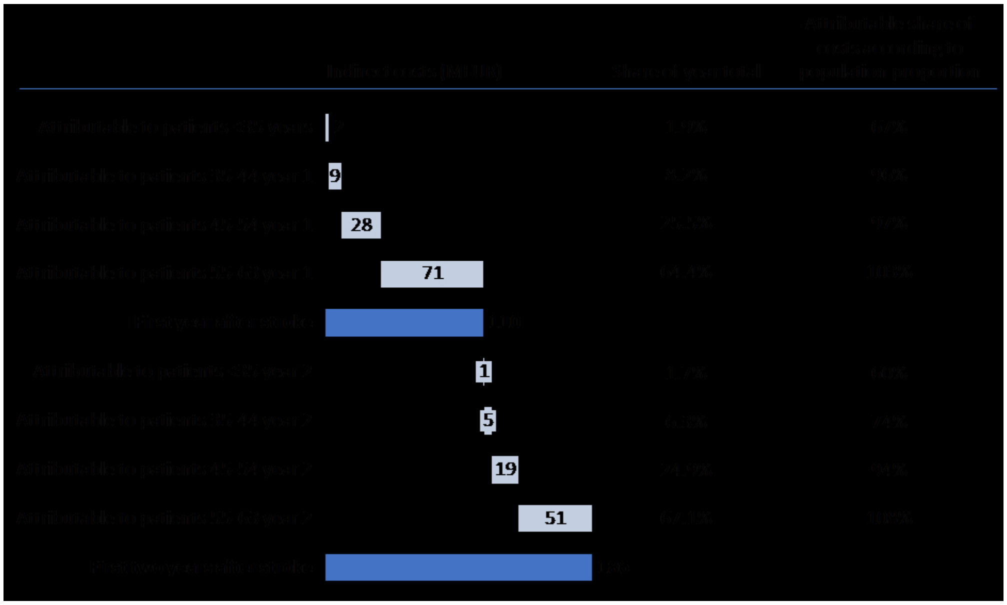

Figure 2. Indirect costs in terms of productivity loss per age group years 1-2 after stroke, based on individual income data from Statistics Sweden. Proportion based on population contribution reflects whether the age-group share of total costs is representative or not (age group under-represented if <100% and over-represented if >100%).

248x148mm (96 x 96 DPI)

Health insurance utilization after ischemic stroke in Sweden: a retrospective cohort study in a system of universal healthcare and social insurance

Journal:	*BMJ Open*
Manuscript ID	bmjopen-2020-043826.R1
Article Type:	Original research
Date Submitted by the Author:	28-Dec-2020

Complete List of Authors:	Willers, Carl; Karolinska Institute, Department of Neurobiology, Care Sciences and Society<br>Westerlind, Emma; Sahlgrenska Academy, Department of Clinical Neuroscience, Institute of Neuroscience and Physiology<br>Borgström, Fredrik; Karolinska Institute, Department of Learning, Informatics, Management and Ethics, Medical Management Centre<br>von Euler, Mia; Karolinska Institute, Department of Medicine Solna; Karolinska Institute, Department of Clinical Science and Education<br>Sunnerhagen, Katharina; Sahlgrenska Academy, Department of Clinical Neuroscience, Institute of Neuroscience and Physiology

**Primary Subject Heading**:	Health economics
Secondary Subject Heading:	Public health, Neurology, Health policy
Keywords:	Stroke < NEUROLOGY, HEALTH ECONOMICS, PUBLIC HEALTH

I, the Submitting Author has the right to grant and does grant on behalf of all authors of the Work (as defined in the below author licence), an exclusive licence and/or a non-exclusive licence for contributions from authors who are: i) UK Crown employees; ii) where BMJ has agreed a CC-BY licence shall apply, and/or iii) in accordance with the terms applicable for US Federal Government officers or employees acting as part of their official duties; on a worldwide, perpetual, irrevocable, royalty-free basis to BMJ Publishing Group Ltd ("BMJ") its licensees and where the relevant Journal is co-owned by BMJ to the co-owners of the Journal, to publish the Work in this journal and any other BMJ products and to exploit all rights, as set out in our licence.

The Submitting Author accepts and understands that any supply made under these terms is made by BMJ to the Submitting Author unless you are acting as an employee on behalf of your employer or a postgraduate student of an affiliated institution which is paying any applicable article publishing charge ("APC") for Open Access articles. Where the Submitting Author wishes to make the Work available on an Open Access basis (and intends to pay the relevant APC), the terms of reuse of such Open Access shall be governed by a Creative Commons licence – details of these licences and which Creative Commons licence will apply to this Work are set out in our licence referred to above.

Other than as permitted in any relevant BMJ Author's Self Archiving Policies, I confirm this Work has not been accepted for publication elsewhere, is not being considered for publication elsewhere and does not duplicate material already published. I confirm all authors consent to publication of this Work and authorise the granting of this licence.

For peer review only

Health insurance utilization after ischemic stroke in Sweden: a retrospective cohort study in a system of universal healthcare and social insurance

Authors: Carl Willers[1], Emma Westerlind[2], Fredrik Borgström[3], Mia von Euler[4], Katharina Sunnerhagen[2]

1. Department of Neurobiology, Care Sciences and Society, Karolinska Institutet, Stockholm, Sweden

2. Department of Clinical Neuroscience, Institute of Neuroscience and Physiology, Sahlgrenska Academy, University of Gothenburg, Gothenburg, Sweden

3. Department of Learning, Informatics, Management and Ethics, Medical Management Centre, Karolinska Institutet, Stockholm, Sweden **and** Quantify Research, Stockholm, Sweden

4. Clinical Pharmacology Unit, Clinical Epidemiology Division, Department of Medicine Solna, Karolinska Institutet, Stockholm, Sweden **and** Department of Clinical Science and Education, Karolinska Institutet Stroke Research Network at Södersjukhuset, Stockholm, Sweden

Correspondence with Carl Willers, carl.willers@ki.se, +46(0)709 96 43 53

For peer review only

Abstract

Background

Stroke is one of the largest single-condition sources of the global burden of non-communicable disease in terms of disability-adjusted life-years and monetary costs, directly as well as indirectly in terms of informal care and productivity loss. The objective was to assess the population afflicted with ischemic stroke in working age in the context of universal healthcare and social insurance; to estimate the levels of absence from work, the indirect costs related to that, and to assess what patient characteristics that are associated.

Methods

This was a retrospective register-based study; all individuals registered with an ischemic stroke during 2008-2011 in seven Swedish regions, covering the largest cities as well as more rural areas, were included. Individual-level data were used to compute net days of sick leave and disability pension, indirect costs due to productivity loss, and to perform regression analysis on net absence from work to assess the associated factors. Costs related to productivity loss were estimated using the Human Capital approach.

Results

Women had significantly fewer net days of sick leave and disability pension than men after multivariate adjustment, and high-income groups had higher levels of sick leave than low-income groups. There were no significant differences for participants regarding educational level, region of birth or civil status. Indirect monetary costs amounted to 17.4 thousand euros per stroke case first year, totaling approximately 169 million euros in Sweden.

Conclusion

The individual's burden of stroke is heavy in terms of morbidity , and the related productivity loss for society is immense. Income-group differences point to a socioeconomic gradient in the utilization of the Swedish social insurance.

Strengths and limitations of this study

- The present study's underlying data are to a large extent extracted from population registries which implies very high coverage rates and hence a close-to-true picture of the actual population
- Sick-leave costs for stroke patients have been calculated before as well as after the stroke event, which adds important information in terms of incremental costs after stroke
- Regression analysis of net days of absence included clinical as well as socioeconomical variables implying broad adjustments and high possibility to understand the actual drivers
- Informal care is costly, and no estimation of informal care has been included in this study due to lack of reliable data
- The actual costs are in fact higher as any cases of sick benefits lasting shorter than 14 days are not registered in the Social Insurance Agency database.

Key words: stroke, social insurance, cost analysis, sick leave

For peer review only

Background

Stroke is a leading cause of long-term disability globally and a resource intensive disease, directly and indirectly [1]. Societal costs in terms of productivity loss are immense and have been for the last decade [2]. A non-negligible proportion of stroke patients is still in working age and these incur substantial direct and indirect costs; stroke and ischemic heart disease are the largest sources of the global burden of non-communicable disease in terms of disability-adjusted life-years [1]. Work disability after stroke is generally of more permanent nature compared to other conditions such as ischemic heart disease [3].

The Swedish social insurance covers individuals who live or work in Sweden; all people who are employed and whose employer pay social contributions to the agency are insured against loss of work-related salary up to a standardized ceiling [4]. The social insurance is considered a social security to inhabitants and companies, and thereby also to society [5]. Better understanding of the situation of health insurance utilization and its drivers could help policy makers, governmental bodies and care providers in their work with designing guidelines, monitoring the development of care and optimizing treatment; evidence of the societal impact of stroke incidence, stroke care and recovery is needed to make good decisions on where to invest in the chain-of-care.

The aim with the present study was to map levels of health insurance utilization in relation to ischemic stroke – the year prior to and the two years after – in a health system characterized by universal healthcare and social insurance. Furthermore, the aim was to estimate the distribution of productivity loss associated with stroke across age groups of working age, and to identify key drivers for utilization of health insurance, for individuals afflicted with ischemic stroke (IS).

Methods

Study population and data sources

Seven Swedish regions, covering approximately 65% of the Swedish working age stroke population, contributed with data to the research database. The study population consisted of persons afflicted with ischemic stroke during 2008-2011 and who were aged 18-63 years at the time of stroke. This to only include adults and to allow for two-year follow-up of health insurance utilization, as the general age of retirement in Sweden historically has been 65 years (64,6-64,7 during 2008-2011 [6]). Participants were included independently of degree of sick leave or disability pension prior to stroke.

Stroke cases were identified based on registration of acute ischemic stroke diagnosis (ICD-10 code I63) in regional administrative systems together with a registration in the Swedish Stroke Register. The latter is a national register for stroke-specific outcomes, reported on optional basis by the caregiver and with a coverage rate of 90% at the time, with aims including to enable research and continuous improvement of stroke care to ensure high-quality and equitable care [7]. Follow-up amounted to at least two years for all participants. Relevant data from research registries were linked on individual level, including data on health insurance (Swedish Social Insurance Agency), socioeconomics and mortality (Statistics Sweden). All data were pseudonymized, and the study was approved by the Regional Ethical Review Board in Stockholm (2013/1541-31/5 and 2016/785-32).

For peer review only

Variable definitions

Productivity loss was defined as the sum of sick leave and/or disability pension (early retirement to some degree) in monetary terms and based on net days (net productivity loss in terms of full working days; sick leave can formally be granted to an extent of 25%, 50%, 75% or 100% per day, and the individual is formally working the remaining proportion with a salary adjusted accordingly). Absence was calculated based on available information from the Swedish Social Insurance Agency; sick leave lasting 14 days or shorter is financed by the employer, and not available in the national registry. Hence only sick leave periods lasting longer than 14 days were included in the analysis.

Information on prior stroke, history of atrial fibrillation and hypertension was based on administrative data, including diagnosis registration, for two years prior to the stroke. Information on dependency for activities of daily living (ADL) was based on data from the Swedish Stroke Register (in need of help with either dressing or toilet visits rendered an ADL value of 1, else 0), as was the computation of the modified Rankin Scale (mRS) which was made in accordance with a published and validated method for approximation [8]. Information on living situation, income, educational level and origin were available from Statistics Sweden. Clinical information on level of consciousness and reperfusion treatment was extracted from the Swedish Stroke Register. Due to low coverage rate of the National Institutes of Health Stroke Severity (NIHSS) in the register (<50% complete for this study population), an approximation of stroke severity was captured via information on level of consciousness at hospital arrival (a version of the Reaction Level Scale which is used in the Swedish Stroke Register) which has been shown to be a good approximation of NIHSS for prediction of e.g. mortality [9].

Calculation of indirect costs

Indirect costs were calculated based on the Human Capital approach [10]; it was assumed that indirect costs after stroke for the individual participant was equivalent to the sum of income the participant would receive if not having a stroke (i.e. based on number of net days of absence and the level of income per day during the year prior to stroke), and the employer contribution that the employer pays for their employees (31.42% of the salary [11]); *[individual income per day] * [individual net days of work absence] * [1.3142]*. Indirect costs related to the first 14 days of the included cases were also estimated and included in the analysis. The authors did however not have any information whether the individual participant was in fact employed at the time of stroke. Information on disposable income was available on individual level and was used as explanatory variable in the regression analysis, then categorized into quartiles. The exchange rate for Swedish krona (SEK) per Euro (EUR) was set to 0.11198, which was the official exchange rate on December 31st, 2011 [12].

Estimation of indirect costs related to productivity loss was made per age group (age spans set prior to the analysis; <35, 35-44, 45-54, 55-63 years of age at time of stroke). The estimate was based on individual-level income data for the study population, available from Statistics Sweden.

For peer review only

Statistical analysis of factors associated to work absence

In addition, multivariate regression analysis was performed, adjusting for a broad range of select factors of relevance (presented under Results). Health insurance utilization was analyzed in terms of sick leave and disability pension (early retirement due to disability) as count variables with negative binomial regression. The reason for treating it as a count variable was that the decision on the total amount of sick leave is iterative and based on a joint agreement between the doctor and the patient done during several occasions. Participants deceased during follow-up were excluded from the analysis.

For peer review only

Results

The study population amounted to 6 637 individuals, whereof 35% women. The study population was split into four age groups (Table 1). The elder the age group, the larger the volume of participants per age group; the youngest age group, <35 years, consisted of 180 individuals, and the eldest age group consisted of 4 174 individuals. The younger the age group, the larger the proportion of women; 52% in the youngest age group, and 33% in the eldest age group. The prevalence of atrial fibrillation and hypertension seemed to increase with higher age, according to registration in medical records. The eldest age group (55-63 years at the time of stroke) versus the youngest (<35 years at stroke) showed a prevalence of 10.9% versus 1.1% (atrial fibrillation) and 54.1% versus 3.9% (hypertension) respectively. Individual income (quartiles 1, 2 and 3) was highest in the group of participants aged 35-44 at the time of stroke. The youngest age group was the most educated in terms of years spent in school with 40,7% having studied for 12 years or more.

For peer review only

Table 1. Baseline characteristics of the study population, per age group

	Age groups				
	**<35 yrs**	**35-44 yrs**	**45-54 yrs**	**55-63 yrs**	
n	180 (3%)	548 (8%)	1 735 (26%)	4 174 (63%)	6637
Sex, proportion women (%)	52	42	37	33	35
ADL dependent (%)	1.7	1.1	1.5	3.0	2.4
Prior stroke, last two years (%)	3.4	4.0	4.3	4.6	4.4
Inpatient care the year prior to stroke (days)	3.25	2.78	3.92	3.81	3.7
Level of consciousness (%)					
Conscious	89.8	94.0	93.4	93.9	93.7
Indolent	5.7	5.2	5.2	4.9	5.0
Unconscious	4.5	0.8	1.4	1.2	1.3
Atrial fibrillation, prior to stroke (%)	1.1	2.9	5.4	10.9	8.5
Hypertension, prior to stroke (%)	3.9	23.0	41.1	54.1	46.8
Reperfusion treatment (thrombolysis or thrombectomy, %)	15.3	14.3	13.0	9.9	11.2
Single household (%)	27.3	21.7	30.8	34.4	32.2
Disposable income (EUR, nominal amount)					
Q1	12 788	16 786	14 401	14 199	14 427
Q2	17 217	24 468	21 646	20 935	21 311
Q3	25 330	32 256	29 787	29 182	29 489
Educational level (%)					
≤9 years	16.95	13.79	27.37	31.04	28.27
10-12 years	42.37	53.31	50	46.41	47.81

For peer review only

≥12 years	40.68	32.9	22.63	22.55	23.92
Born outside the EU (%)	10.0	15.0	15.6	8.7	11.1
Sick leave the year prior to stroke (net days)	17.7	19.1	18.4	17.3	17.7
Disability pension the year prior to stroke (net days)	25.0	34.5	74.0	108.7	91.2

Note: level of consciousness at hospital arrival is subject to judgement by responsible clinician. Disposable income is equivalent to total income minus taxes. The categories of educational level stand for the Swedish equivalents of high school, secondary school and post-secondary education.

For peer review only

The individual levels of absence from work due to sickness the year prior to stroke varied between the age groups (Figure 1). The youngest age groups showed average absence of 42.7 and 53.6 net days respectively, whilst individuals in ages 45-54 at stroke presented with 92.4 net days of absence the year prior to stroke. Individuals aged 55-63 at stroke had 126.0 net days of absence the year prior to stroke. The higher the age group, the higher the ratio of disability pension; the levels of net days prior to stroke were made up of 59% disability pension (<35 years), 64% disability pension (35-44 years), 80% disability pension (45-54 years) and 86% disability pension (55-63 years) respectively (the remaining proportion of net days regarded sickness benefit).

During the first year after stroke, levels of absence increased significantly from the year before, but overall, levels were on more similar levels across the different age groups; 153.1 (<35 years at stroke), 175.8 (35-44 years at stroke), 194.8 (45-54 years at stroke), and 211.8 (55-63 years at stroke) net days respectively. The increase between the year prior to stroke versus after stroke was highest in the youngest age group (3.6 times higher) and lowest in the eldest age group (1.7 times higher).

Levels of absence the second year after stroke were significantly lower than levels the first year after stroke. The largest relative drop in absence was found in the second youngest age group (35-44 years) and amounted to -33.5%. The age group with the lowest relative increase in the second year compared to the year prior to stroke was the eldest age group with 41% more net days.

Figure 1. Average health insurance levels utilized per individual, before and after stroke per age group: net days of sick leave and/or disability pension.

For peer review only

The productivity loss amounted to 110 Million Euros during the first year after stroke (Figure 2). The indirect costs were 27% lower during the second year after stroke and amounted to 76 Million Euros. The age-group share of total was similar during the first and second year although the second youngest age group was relatively less costly during the second year after stroke.

The average indirect cost per participant per year during the first and second year amounted to 11.6 kEUR and 7.3 kEUR for the youngest age group, 16.6 kEUR and 9.0 kEUR for participants aged 35-44, 16.8 kEUR and 11.5 kEUR for participants aged 45-54, and 18.0 kEUR and 13.2 kEUR for participants aged 55-63 at the time of stroke. On average across all four age groups, indirect monetary costs amounted to 17.4 kEUR per stroke case first year and 12.2 kEUR second year.

Figure 2. Indirect costs in terms of productivity loss per age group years 1-2 after stroke, based on individual income data from Statistics Sweden. Proportion based on population contribution reflects whether the age-group share of total costs is representative or not (age group under-represented if <100% and over-represented if >100%).

When adjusting for clinical and sociodemographic factors potentially associated with levels of work absence after stroke, shown in Table 2, belonging to a certain age group was not significantly associated to differences in absence from work due to sick leave or disability pension.

Women had significantly fewer net days of absence during the year after stroke compared to men, after multivariate adjustments. Participants in the lowest (first) income quartile had significantly lower absence levels compared to participants in the second, third and fourth quartile. There were no significant differences for participants regarding educational level, region of birth or civil status.

For peer review only

Table 2. All covariates used in the multivariate regression analysis of post-stroke health insurance utilization (coefficients and 95% CI from regression analysis): net days of absence (sick leave and/or disability pension)

	**Coefficient**	**95% CI**		**p value**
Sex, women	-0.085	-0.167	-0.004	0.040
Age				
<35	[reference]			
35-44	0.144	-0.114	0.402	0.274
45-54	0.158	-0.081	0.397	0.196
55-63	0.150	-0.085	0.386	0.211
ADL dependent	0.020	-0.299	0.340	0.902
Prior stroke (last two years)	-0.059	-0.257	0.140	0.561
Inpatient care the year prior to stroke	0.001	-0.002	0.004	0.562
Net days of absence the year prior to stroke (sick leave and/or disability pension)	0.003	0.003	0.003	0.000
Living situation				
At home	[reference]			
At home with home care	-0.067	-0.321	0.188	0.607
At special housing	-0.067	-0.436	0.303	0.724
Level of consciousness				
Conscious	[reference]			
Indolent	0.398	0.211	0.586	0.000
Unconscious	0.353	-0.083	0.790	0.113

Single household	0.013	-0.084	0.109	0.794
Disposable income				
Quartile 1	[reference]			
Quartile 2	0.309	0.201	0.417	0.000
Quartile 3	0.417	0.302	0.532	0.000
Quartile 4	0.218	0.097	0.340	0.000
Educational level				
<9 years	[reference]			
10-12 years	0.022	-0.068	0.112	0.632
>12 years	-0.079	-0.190	0.031	0.161
Born outside of the EU	0.033	-0.092	0.157	0.609
Marital status				
Married	[reference]			
Never married	0.076	-0.028	0.180	0.153
Divorced	0.030	-0.075	0.134	0.575
Widowed	-0.106	-0.366	0.154	0.425

only

Discussion

The burden associated with stroke is immense – in terms of Health-Related Quality of Life [1] as well as money [13-15]. In addition to severe personal suffering, stroke affects society by putting a significant burden on direct healthcare resources but also by indirect, major and long-lasting productivity loss. According to the present study, first-year indirect costs in terms of productivity loss and employer contributions amount to on average 15 400 Euros per stroke case in younger working ages (up to 44 years) and to 17 600 Euros per stroke case in the older working ages (45-63 years at stroke). Second-year indirect monetary costs amounted to on average 8 600 Euros per stroke case in the younger group and to 12 700 Euros per stroke case in the older group. The first-year indirect monetary costs for stroke in the present study totaled 110 Million Euros. Given that almost two thirds of the Swedish stroke cases were the basis for analysis and that both urban and rural areas are represented, it is likely not unfair to make an extrapolation for all of Sweden; assuming equivalent incidence and equivalent levels of income for all of Sweden renders an estimate of approximately 169 Million Euros annually – including only the first year after stroke. The costs are not limited to the first or second year (indirect monetary costs of 76 MEUR for the study population, estimated to 117 MEUR for the full Swedish population), but expected to last several years after the stroke event [16]. Information on monetary productivity loss per year due to stroke in other countries is scarce, as shown in previous literature review [17], but significantly lower levels than what was found in this study has been reported (e.g. £ 2 175 per case for UK [18]). Indirect costs related to stroke incidence in Sweden have been assessed in previous studies and found to be a minor proportion of the total cost, amounting to 1.99-5.94 kEUR per stroke first year [13-15]. These studies did however include persons of all ages afflicted with stroke, and likely the indirect costs are significantly higher for the group in general working age as included in the present study. The estimate of total indirect costs of stroke in Sweden in this study (169 MEUR first year and 117 MEUR second year) is on par with a 2020 published estimate of 122 MEUR (productivity loss due to morbidity) [19].

Levels of sick leave the year prior to stroke were similar between the age groups (17.3-19.1 net days), whilst absence due to disability pension the year prior to stroke varied significantly (25.0-108.7 net days); the older the age group, the higher absolute and relative levels of disability pension. The levels of net absence during the year prior to stroke points at the fact that participants active on the labor market were more afflicted with disease than the average general population; pre-stroke levels of sick leave (absence excluding disability pension) in the study population amounted to 17.7 net days (average of all age groups) whilst the national average for the same time period amounted to 6.0 net days (2010) [20]. The patterns are the same looking at the health insurance as a whole; total absence in the study population the year prior to stroke amounted to more than three times the total absence in the general population for the study population (109.0 net days compared to 29.5 net days 2010 [20]). The proportion of women over the different age groups differed; the younger the age group, the higher the proportion of women. Higher proportion of women in younger age categories may be related to impacted hormone levels from contraceptives or pregnancy, e.g. changed balance in coagulation factors or hypertensive crisis [21].

Sex and level of consciousness at hospital arrival were significantly associated with the level of absence the year after stroke; women were less absent on sick leave and/or disability pension as were participants who were conscious at hospital arrival compared to those who were indolent. The finding that female sex was significantly associated with lower levels of absence the year after stroke stood out compared to the general population; according to the Social Insurance Agency, women have higher risk for being on sick leave, a difference which has continued to increase since 2010 [22]. Sick leave figures in public reports are often presented without multivariate adjustments which was

For peer review only

the case in the present study. Furthermore, disposable income showed strong association with absence after stroke; participants in the lowest income group had significantly lower levels of absence compared to all other income groups.

Absence the year prior to stroke was positively correlated with absence the year after stroke. Interestingly, when this baseline characteristic was not taken into account, several socioeconomic indicators showed significant association with absence the year after stroke, including educational level, birth region and civil status.

The present study's underlying data are to a large extent extracted from population registries which implies very high coverage rates and hence a close-to-true picture of the actual population, and sick-leave costs for stroke patients have been calculated before as well as after the stroke event, which adds important information in terms of incremental costs after stroke. The actual costs are in fact higher as any cases of sick benefits lasting shorter than 14 days are not registered in the Social Insurance Agency database. Furthermore, informal care is costly, and no estimation of informal care has been included in this study due to lack of reliable data. During 2008, significant changes were implemented in the Swedish health insurance to limit the length of time possible to receive support for loss of income due to disability. These changes were implemented at the beginning of the time period assessed in the present study and could be expected to influence the estimation of costs equivalently over the time period studied [23].

The fact that low-income individuals had fewer net days of sick leave is important to note as this points to a socioeconomic gradient in the utilization of the general health insurance. As health equity is a topic treated in the Swedish healthcare act it is particularly relevant to evaluate whether the distribution of governmental funding is actually equitable. Disposable income is an example of a factor that should not be associated to social insurance utilization after consideration has been taken to medical factors. There are several different factors that may impact and drive that gradient, but it is likely that factors such as higher general dependence on income in lower-income professions and higher general knowledge regarding the social insurance system in higher-income groups are of relevance to explain this gradient [24]. It is also likely that underlying themes such as knowledge of and ability to pursue primary and secondary stroke prevention is asymmetrically distributed across e.g. income groups.

Conclusions

Individuals that are afflicted with stroke in working age have a significantly higher pre-stroke level of health insurance (sick leave or disability pension) utilization than the average in Sweden. The monetary burden of stroke is very high; the first-year indirect costs for an individual in working age amounts to more than one third of the GDP per capita (reference year 2011). There is possibly a socioeconomic gradient in the utilization of Swedish health insurance among stroke patients; high-income individuals generally had higher levels of sick leave than did low-income individuals, while simultaneously adjusting for other relevant factors on patient level. Continuous assessments of the social insurance utilization among stroke patients – as well as for other conditions – by governmental institutions are encouraged, to follow up on the equity in its design and practical implications.

For peer review only

List of abbreviations

ADL – activities of daily living

EU – European Union

GDP – Gross Domestic Product

IS – ischemic stroke

EUR - Euros

Contributorship

CW and KSS conceived the idea to the study. CW performed the analyses and drafted the manuscript. CW, EW, FB, MvE and KSS contributed to the final version and approved the submitted version.

Competing interests

None declared.

Funding

This study was supported by grants from the Swedish Research Council (VR2017-00946), the Swedish Heart and Lung Foundation, the Swedish Brain Foundation, the Promobilia Foundation, the Norrbacka-Eugenia Foundation as well as by grants from the Swedish state under an agreement between the Swedish government and the county councils, the ALF agreement (ALFGBG-718711).

Data sharing

The dataset generated and analysed during the current study is not publicly available due to Swedish law; access to the dataset was given to the researchers under the condition of timely valid ethical approval. Additional information regarding the availability is available from the corresponding author upon request.

Acknowledgements

We are grateful to the Swedish Stroke Register (www.riksstroke.org) and the different healthcare regions for providing data for this study. We also wish to thank all patients, caregivers, reporting units, and coordinators in Riksstroke as well as Riksstroke steering committee.

Patient and Public Invlovement

It was not appropriate or possible to involve patients or the public in the design, or conduct, or reporting, or dissemination plans of our research.

For peer review only

References

1. *Global, regional, and national burden of stroke, 1990-2016: a systematic analysis for the Global Burden of Disease Study 2016.* Lancet Neurol, 2019. **18**(5): p. 439-458 GBD Stroke Collaborators.
2. Luengo-Fernandez, R., et al., *Economic burden of stroke across Europe: A population-based cost analysis.* European Stroke Journal, 2019: p. 2396987319883160.
3. Ervasti, J., et al., *Permanent work disability before and after ischaemic heart disease or stroke event: a nationwide population-based cohort study in Sweden.* BMJ Open, 2017. **7**(9): p. e017910.
4. *The Swedish social insurance during 2018 (Socialförsäkringen i siffror 2018).* 2018, The Swedish Social Insurance Agency: Stockholm.
5. *About the Swedish social insurance*, T.S.S.I. Agency, Editor. 2019.
6. *Average age of retirement and exit from the Swedish labor market (Medelpensioneringsålder och utträdesålder)*. 2016, The Swedish Pensions Agency.
7. Söderholm, A., et al., *Validation of Hospital Performance Measures of Acute Stroke Care Quality. Riksstroke, the Swedish Stroke Register.* Neuroepidemiology, 2016. **46**(4): p. 229-34.
8. Eriksson, M., et al., *Assessment of functional outcome in a national quality register for acute stroke: can simple self-reported items be transformed into the modified Rankin Scale?* Stroke, 2007. **38**(4): p. 1384-6.
9. Bray, B.D., et al., *Derivation and external validation of a case mix model for the standardized reporting of 30-day stroke mortality rates.* Stroke, 2014. **45**(11): p. 3374-80.
10. Drummond, M.F., *Methods for the economic evaluation of health care programmes*. 3rd edition ed. 2005, Oxford: Oxford University Press.
11. *Size of employer contributions*. [cited 2019 25 October]; Description of employer contribution for persons in general working age and its components:[Available from: https://www4.skatteverket.se/rattsligvagledning/edition/2014.14/1333.html.
12. *Oanda Currency Converter*. 2019 [cited 2019 November 14]; Available from: https://www1.oanda.com/currency/converter/.
13. Lekander, I., et al., *Relationship between functional disability and costs one and two years post stroke.* PLoS One, 2017. **12**(4): p. e0174861.
14. Ghatnekar, O., et al., *Cost of stroke in Sweden: an incidence estimate.* Int J Technol Assess Health Care, 2004. **20**(3): p. 375-80.
15. Ghatnekar, O., et al., *Costs for stroke in Sweden 2009 and developments since 1997.* Int J Technol Assess Health Care, 2014. **30**(2): p. 203-9.
16. Gloede, T.D., et al., *Long-term costs of stroke using 10-year longitudinal data from the North East Melbourne Stroke Incidence Study.* Stroke, 2014. **45**(11): p. 3389-94.
17. Luengo-Fernandez, R., A.M. Gray, and P.M. Rothwell, *Costs of stroke using patient-level data: a critical review of the literature.* Stroke, 2009. **40**(2): p. e18-23.
18. Saka, O., A. McGuire, and C. Wolfe, *Cost of stroke in the United Kingdom.* Age Ageing, 2009. **38**(1): p. 27-32.
19. Luengo-Fernandez, R., et al., *Economic burden of stroke across Europe: A population-based cost analysis.* Eur Stroke J, 2020. **5**(1): p. 17-25.
20. *Social Insurance Report 2011:6*. 2011, The Swedish Social Insurance Agency.
21. Cordonnier, C., et al., *Stroke in women - from evidence to inequalities.* Nat Rev Neurol, 2017. **13**(9): p. 521-532.
22. *Social Insurance Report 2018:2*. 2018, The Swedish Social Insurance Agency.
23. *Follow-up report of the health insurance reform, reference number 037795-2011 (Försäkringskassans uppföljning av sjukförsäkringsreformen – delredovisning)*. 2011, The Swedish Social Insurance Agency.

For peer review only

24. *Insured but not reimbursed - a report on the under-utilization of the union-based health insurance (Försäkrad men utan ersättning - en granskning av underutnyttjandet av den kollektivavtalade sjukförsäkringen); 2018:7*. 2018, The Swedish Social Insurance Inspectorate.

For peer review only

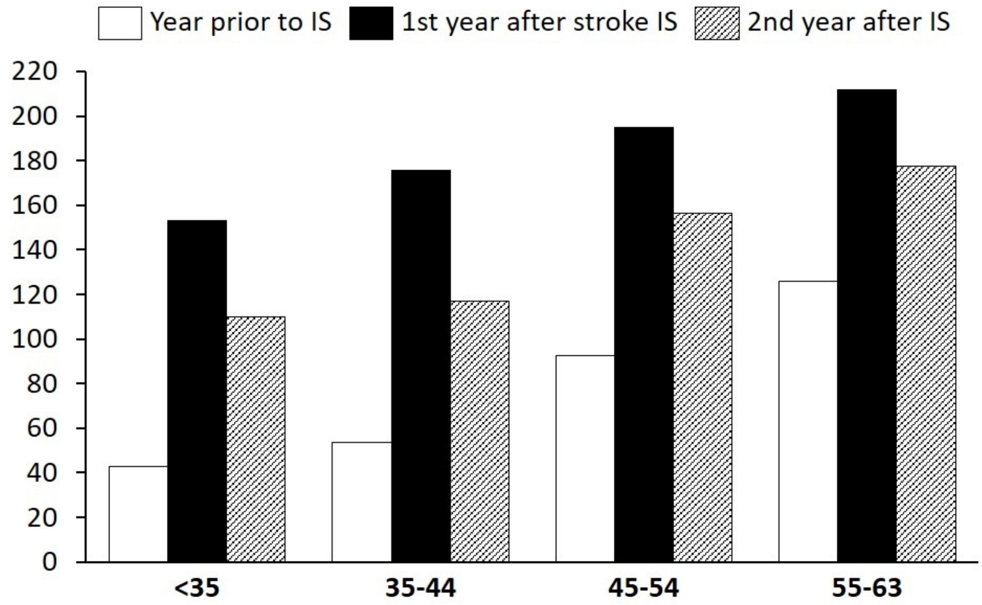

Figure 1. Average health insurance levels utilized per individual, before and after stroke per age group: net days of sick leave and/or disability pension.

80x51mm (300 x 300 DPI)

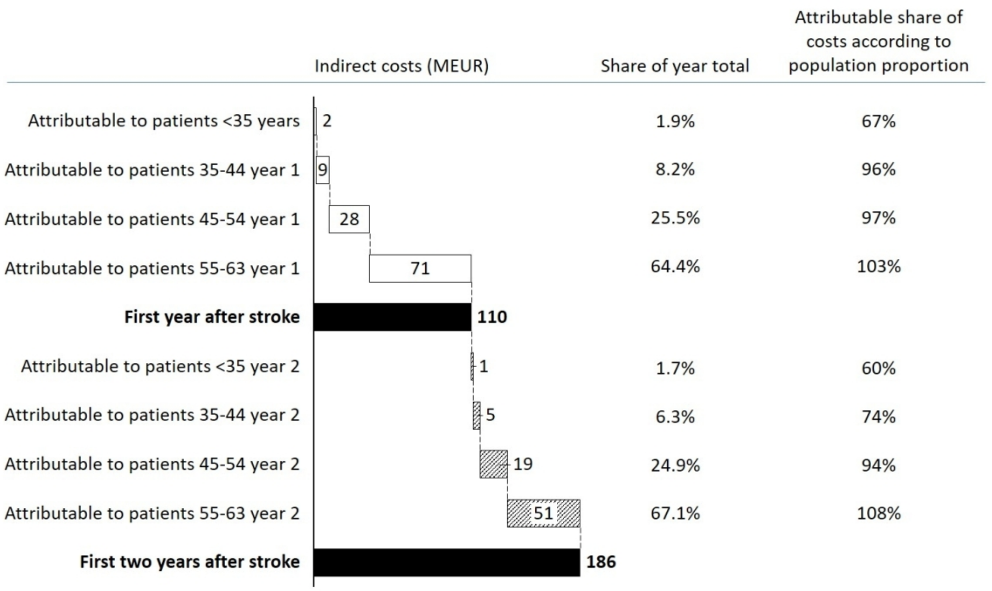

Figure 2. Indirect costs in terms of productivity loss per age group years 1-2 after stroke, based on individual income data from Statistics Sweden. Proportion based on population contribution reflects whether the age-group share of total costs is representative or not (age group under-represented if <100% and over-represented if >100%).

123x74mm (300 x 300 DPI)

For peer review only - http://bmjopen.bmj.com/site/about/guidelines.xhtml

STROBE Statement—checklist of items that should be included in reports of observational studies

	Item No.	Recommendation	Page No.
**Title and abstract**	1	(*a*) Indicate the study's design with a commonly used term in the title or the abstract	1
		(*b*) Provide in the abstract an informative and balanced summary of what was done and what was found	2
**Introduction**			
Background/rationale	2	Explain the scientific background and rationale for the investigation being reported	3
Objectives	3	State specific objectives, including any prespecified hypotheses	3
**Methods**			
Study design	4	Present key elements of study design early in the paper	3
Setting	5	Describe the setting, locations, and relevant dates, including periods of recruitment, exposure, follow-up, and data collection	3
Participants	6	(*a*) *Cohort study*—Give the eligibility criteria, and the sources and methods of selection of participants. Describe methods of follow-up<br>*Case-control study*—Give the eligibility criteria, and the sources and methods of case ascertainment and control selection. Give the rationale for the choice of cases and controls<br>*Cross-sectional study*—Give the eligibility criteria, and the sources and methods of selection of participants	3-5
		(*b*) *Cohort study*—For matched studies, give matching criteria and number of exposed and unexposed<br>*Case-control study*—For matched studies, give matching criteria and the number of controls per case	
Variables	7	Clearly define all outcomes, exposures, predictors, potential confounders, and effect modifiers. Give diagnostic criteria, if applicable	4
Data sources/ measurement	8*	For each variable of interest, give sources of data and details of methods of assessment (measurement). Describe comparability of assessment methods if there is more than one group	3-4
Bias	9	Describe any efforts to address potential sources of bias	4-5
Study size	10	Explain how the study size was arrived at	3

Continued on next page

For peer review only

Quantitative variables	11	Explain how quantitative variables were handled in the analyses. If applicable, describe which groupings were chosen and why	4-5
Statistical methods	12	(*a*) Describe all statistical methods, including those used to control for confounding	4-5
		(*b*) Describe any methods used to examine subgroups and interactions	4-5
		(*c*) Explain how missing data were addressed	5
		(*d*) *Cohort study*—If applicable, explain how loss to follow-up was addressed<br>*Case-control study*—If applicable, explain how matching of cases and controls was addressed<br>*Cross-sectional study*—If applicable, describe analytical methods taking account of sampling strategy	
		(*e*) Describe any sensitivity analyses	
Participants	13*	(a) Report numbers of individuals at each stage of study—eg numbers potentially eligible, examined for eligibility, confirmed eligible, included in the study, completing follow-up, and analysed	3, 7
		(b) Give reasons for non-participation at each stage	3, 5
		(c) Consider use of a flow diagram	
Descriptive data	14*	(a) Give characteristics of study participants (eg demographic, clinical, social) and information on exposures and potential confounders	7-8
		(b) Indicate number of participants with missing data for each variable of interest	7
		(c) *Cohort study*—Summarise follow-up time (eg, average and total amount)	3
Outcome data	15*	*Cohort study*—Report numbers of outcome events or summary measures over time	7-10
		Case-control study—Report numbers in each exposure category, or summary measures of exposure	
		Cross-sectional study—Report numbers of outcome events or summary measures	
Main results	16	(*a*) Give unadjusted estimates and, if applicable, confounder-adjusted estimates and their precision (eg, 95% confidence interval). Make clear which confounders were adjusted for and why they were included	7-10
		(*b*) Report category boundaries when continuous variables were categorized	7
		(*c*) If relevant, consider translating estimates of relative risk into absolute risk for a meaningful time period	

Continued on next page

For peer review only

Other analyses	17	Report other analyses done—eg analyses of subgroups and interactions, and sensitivity analyses	10
Key results	18	Summarise key results with reference to study objectives	9-10
Limitations	19	Discuss limitations of the study, taking into account sources of potential bias or imprecision. Discuss both direction and magnitude of any potential bias	14
Interpretation	20	Give a cautious overall interpretation of results considering objectives, limitations, multiplicity of analyses, results from similar studies, and other relevant evidence	13-14
Generalisability	21	Discuss the generalisability (external validity) of the study results	13
**Other information**			
Funding	22	Give the source of funding and the role of the funders for the present study and, if applicable, for the original study on which the present article is based	15

*Give information separately for cases and controls in case-control studies and, if applicable, for exposed and unexposed groups in cohort and cross-sectional studies.

Note: An Explanation and Elaboration article discusses each checklist item and gives methodological background and published examples of transparent reporting. The STROBE checklist is best used in conjunction with this article (freely available on the Web sites of PLoS Medicine at http://www.plosmedicine.org/, Annals of Internal Medicine at http://www.annals.org/, and Epidemiology at http://www.epidem.com/). Information on the STROBE Initiative is available at www.strobe-statement.org.

For peer review only

BMJ Open

Health insurance utilization after ischemic stroke in Sweden: a retrospective cohort study in a system of universal healthcare and social insurance

Journal:	*BMJ Open*
Manuscript ID	bmjopen-2020-043826.R2
Article Type:	Original research
Date Submitted by the Author:	22-Feb-2021
Complete List of Authors:	Willers, Carl; Karolinska Institute, Department of Neurobiology, Care Sciences and Society<br>Westerlind, Emma; Sahlgrenska Academy, Department of Clinical Neuroscience, Institute of Neuroscience and Physiology<br>Borgström, Fredrik; Karolinska Institute, Department of Learning, Informatics, Management and Ethics, Medical Management Centre<br>von Euler, Mia; Karolinska Institute, Department of Medicine Solna; Karolinska Institute, Department of Clinical Science and Education<br>Sunnerhagen, Katharina; Sahlgrenska Academy, Department of Clinical Neuroscience, Institute of Neuroscience and Physiology
**Primary Subject Heading**:	Health economics
Secondary Subject Heading:	Public health, Neurology, Health policy
Keywords:	Stroke < NEUROLOGY, HEALTH ECONOMICS, PUBLIC HEALTH

SCHOLARONE™
Manuscripts

I, the Submitting Author has the right to grant and does grant on behalf of all authors of the Work (as defined in the below author licence), an exclusive licence and/or a non-exclusive licence for contributions from authors who are: i) UK Crown employees; ii) where BMJ has agreed a CC-BY licence shall apply, and/or iii) in accordance with the terms applicable for US Federal Government officers or employees acting as part of their official duties; on a worldwide, perpetual, irrevocable, royalty-free basis to BMJ Publishing Group Ltd ("BMJ") its licensees and where the relevant Journal is co-owned by BMJ to the co-owners of the Journal, to publish the Work in this journal and any other BMJ products and to exploit all rights, as set out in our licence.

The Submitting Author accepts and understands that any supply made under these terms is made by BMJ to the Submitting Author unless you are acting as an employee on behalf of your employer or a postgraduate student of an affiliated institution which is paying any applicable article publishing charge ("APC") for Open Access articles. Where the Submitting Author wishes to make the Work available on an Open Access basis (and intends to pay the relevant APC), the terms of reuse of such Open Access shall be governed by a Creative Commons licence – details of these licences and which Creative Commons licence will apply to this Work are set out in our licence referred to above.

Other than as permitted in any relevant BMJ Author's Self Archiving Policies, I confirm this Work has not been accepted for publication elsewhere, is not being considered for publication elsewhere and does not duplicate material already published. I confirm all authors consent to publication of this Work and authorise the granting of this licence.

For peer review only

Health insurance utilization after ischemic stroke in Sweden: a retrospective cohort study in a system of universal healthcare and social insurance

Authors: Carl Willers[1], Emma Westerlind[2], Fredrik Borgström[3], Mia von Euler[4], Katharina Sunnerhagen[2]

1. Department of Neurobiology, Care Sciences and Society, Karolinska Institutet, Stockholm, Sweden

2. Department of Clinical Neuroscience, Institute of Neuroscience and Physiology, Sahlgrenska Academy, University of Gothenburg, Gothenburg, Sweden

3. Department of Learning, Informatics, Management and Ethics, Medical Management Centre, Karolinska Institutet, Stockholm, Sweden **and** Quantify Research, Stockholm, Sweden

4. Clinical Pharmacology Unit, Clinical Epidemiology Division, Department of Medicine Solna, Karolinska Institutet, Stockholm, Sweden **and** Department of Clinical Science and Education, Karolinska Institutet Stroke Research Network at Södersjukhuset, Stockholm, Sweden

Correspondence with Carl Willers, carl.willers@ki.se, +46(0)709 96 43 53

For peer review only

Abstract

Background

Stroke is one of the largest single-condition sources of the global burden of non-communicable disease in terms of disability-adjusted life-years and monetary costs, directly as well as indirectly in terms of informal care and productivity loss. The objective was to assess the population afflicted with ischemic stroke in working age in the context of universal healthcare and social insurance; to estimate the levels of absence from work, the indirect costs related to that, and to assess what patient characteristics that are associated.

Methods

This was a retrospective register-based study; all individuals registered with an ischemic stroke during 2008-2011 in seven Swedish regions, covering the largest cities as well as more rural areas, were included. Individual-level data were used to compute net days of sick leave and disability pension, indirect costs due to productivity loss, and to perform regression analysis on net absence from work to assess the associated factors. Costs related to productivity loss were estimated using the Human Capital approach.

Results

Women had significantly fewer net days of sick leave and disability pension than men after multivariable adjustment, and high-income groups had higher levels of sick leave than low-income groups. There were no significant differences for participants regarding educational level, region of birth or civil status. Indirect monetary costs amounted to 17.4 thousand euros per stroke case first year, totaling approximately 169 million euros in Sweden.

Conclusion

The individual's burden of stroke is heavy in terms of morbidity, and the related productivity loss for society is immense. Income-group differences point to a socioeconomic gradient in the utilization of the Swedish social insurance.

Strengths and limitations of this study

- The present study's underlying data are to a large extent extracted from population registries which implies very high coverage rates and hence a close-to-true picture of the actual population
- Sick-leave costs for stroke patients have been calculated before as well as after the stroke event, which adds important information in terms of incremental costs after stroke
- Regression analysis of net days of absence included clinical as well as socioeconomical variables implying broad adjustments and high possibility to understand the actual drivers
- Informal care is costly, and no estimation of informal care has been included in this study due to lack of reliable data
- The actual costs are in fact higher as any cases of sick benefits lasting shorter than 14 days are not registered in the Social Insurance Agency database.

Key words: stroke, social insurance, cost analysis, sick leave

For peer review only

Background

Stroke is a leading cause of long-term disability globally and a resource intensive disease, directly and indirectly [1]. Societal costs in terms of productivity loss are immense and have been for the last decade [2]. A non-negligible proportion of stroke patients is still in working age and these incur substantial direct and indirect costs; stroke and ischemic heart disease are the largest sources of the global burden of non-communicable disease in terms of disability-adjusted life-years [1]. Work disability after stroke is generally of more permanent nature compared to other conditions such as ischemic heart disease [3].

The Swedish social insurance covers individuals who live or work in Sweden; all people who are employed and whose employer pay social contributions to the agency are insured against loss of work-related salary up to a standardized ceiling [4]. The social insurance is considered a social security to inhabitants and companies, and thereby also to society [5]. Better understanding of the situation of health insurance utilization and its drivers could help policy makers, governmental bodies and care providers in their work with designing guidelines, monitoring the development of care and optimizing treatment; evidence of the societal impact of stroke incidence, stroke care and recovery is needed to make good decisions on where to invest in the chain-of-care.

The aim with the present study was to map levels of health insurance utilization in relation to ischemic stroke – the year prior to and the two years after – in a health system characterized by universal healthcare and social insurance. Furthermore, the aim was to estimate the distribution of productivity loss associated with stroke across age groups of working age, and to identify key drivers for utilization of health insurance, for individuals afflicted with ischemic stroke (IS).

Methods

Study population and data sources

Seven Swedish regions, covering approximately 65% of the Swedish working age stroke population, contributed with data to the research database. The study population consisted of persons afflicted with ischemic stroke during 2008-2011 and who were aged 18-63 years at the time of stroke. This to only include adults and to allow for two-year follow-up of health insurance utilization, as the general age of retirement in Sweden historically has been 65 years (64,6-64,7 during 2008-2011 [6]). Participants were included independently of degree of sick leave or disability pension prior to stroke.

Stroke cases were identified based on registration of acute ischemic stroke diagnosis (ICD-10 code I63) in regional administrative systems together with a registration in the Swedish Stroke Register. The regional administrative systems include information on healthcare consumption and registered diagnoses. The Swedish Stroke Register is a national register for stroke-specific outcomes, reported on optional basis by the caregiver and with a coverage rate of 90% at the time, with aims including to enable research and continuous improvement of stroke care to ensure high-quality and equitable care [7]. Follow-up amounted to at least two years for all participants. Relevant data from research registries were linked on individual level, including data on health insurance (Swedish Social Insurance Agency), socioeconomics and mortality (Statistics Sweden). Data sources are specified for each variable in Table 1.

All data were pseudonymized, and the study was approved by the Regional Ethical Review Board in Stockholm (2013/1541-31/5 and 2016/785-32).

For peer review only

Variable definitions

Productivity loss was defined as the sum of sick leave and/or disability pension (early retirement to some degree) in monetary terms and based on net days (net productivity loss in terms of full working days; sick leave can formally be granted to an extent of 25%, 50%, 75% or 100% per day, and the individual is formally working the remaining proportion with a salary adjusted accordingly). Absence was calculated based on available information from the Swedish Social Insurance Agency; sick leave lasting 14 days or shorter is financed by the employer, and not available in the national registry. Hence only sick leave periods lasting longer than 14 days were included in the analysis.

Information on prior stroke, history of atrial fibrillation and hypertension was based on administrative data, including diagnosis registration, for two years prior to the stroke. Information on dependency for activities of daily living (ADL) was based on data from the Swedish Stroke Register (in need of help with either dressing or toilet visits rendered an ADL value of 1, else 0), as was the computation of the modified Rankin Scale (mRS) which was made in accordance with a published and validated method for approximation [8]. Information on living situation, income, educational level and origin were available from Statistics Sweden. Clinical information on level of consciousness and reperfusion treatment was extracted from the Swedish Stroke Register. Due to low coverage rate of the National Institutes of Health Stroke Severity (NIHSS) in the register (<50% complete for this study population), an approximation of stroke severity was captured via information on level of consciousness at hospital arrival (a version of the Reaction Level Scale which is used in the Swedish Stroke Register) which has been shown to be a good approximation of NIHSS for prediction of e.g. mortality [9].

Calculation of indirect costs

Indirect costs were calculated based on the Human Capital approach [10]; it was assumed that indirect costs after stroke for the individual participant was equivalent to the sum of income the participant would receive if not having a stroke (i.e. based on number of net days of absence and the level of income per day during the year prior to stroke), and the employer contribution that the employer pays for their employees (31.42% of the salary [11]); *[individual income per day] * [individual net days of work absence] * [1.3142]*. Indirect costs related to the first 14 days of the included cases were also estimated and included in the analysis. The authors did however not have any information whether the individual participant was in fact employed at the time of stroke. Information on disposable income was available on individual level and was used as explanatory variable in the regression analysis, then categorized into quartiles. The exchange rate for Swedish krona (SEK) per Euro (EUR) was set to 0.11198, which was the official exchange rate on December 31st, 2011 [12].

Estimation of indirect costs related to productivity loss was made per age group (age spans set prior to the analysis; <35, 35-44, 45-54, 55-63 years of age at time of stroke). The estimate was based on individual-level income data for the study population, available from Statistics Sweden.

Statistical analysis of factors associated to work absence

In addition, multivariable regression analysis was performed, adjusting for a broad range of patient characteristics of relevance (presented in Table 2). Health insurance utilization was analyzed; net days of sick leave and disability pension (early retirement due to disability) were used as dependent variable, treated as count variable, and analyzed with negative binomial regression. The reason for treating it as a count variable was that the decision on the total amount of sick leave is iterative and based on a joint agreement between the doctor and the patient done during several occasions. Participants deceased during follow-up were excluded from the analysis.

For peer review only

Results

Study population

The study population amounted to 6 637 individuals, whereof 35% women. The study population was split into four age groups (Table 1). The elder the age group, the larger the volume of participants per age group; the youngest age group, <35 years, consisted of 180 individuals, and the eldest age group consisted of 4 174 individuals. The younger the age group, the larger the proportion of women; 52% in the youngest age group, and 33% in the eldest age group. The prevalence of atrial fibrillation and hypertension seemed to increase with higher age, according to registration in medical records. The eldest age group (55-63 years at the time of stroke) versus the youngest (<35 years at stroke) showed a prevalence of 10.9% versus 1.1% (atrial fibrillation) and 54.1% versus 3.9% (hypertension) respectively. Individual income (quartiles 1, 2 and 3) was highest in the group of participants aged 35-44 at the time of stroke. The youngest age group was the most educated in terms of years spent in school with 40,7% having studied for 12 years or more.

For peer review only

Table 1. Baseline characteristics of the study population, per age group

	Age groups					Data source
	<35 yrs	35-44 yrs	45-54 yrs	55-63 yrs		
n	180 (3%)	548 (8%)	1 735 (26%)	4 174 (63%)	6637	
Sex, proportion women (%)	52	42	37	33	35	Statistics Sweden
ADL dependent (%)	1.7	1.1	1.5	3.0	2.4	Swedish Stroke Register
Prior stroke, last two years (%)	3.4	4.0	4.3	4.6	4.4	Regional administrative systems
Inpatient care the year prior to stroke (days)	3.25 (StdErr 1.03)	2.78 (StdErr 0.42)	3.92 (StdErr 0.43)	3.81 (StdErr 0.19)	3.7	Regional administrative systems
Level of consciousness (%)						Swedish Stroke Register
Conscious	89.8	94.0	93.4	93.9	93.7	
Indolent	5.7	5.2	5.2	4.9	5.0	
Unconscious	4.5	0.8	1.4	1.2	1.3	
Atrial fibrillation, prior to stroke (%)	1.1	2.9	5.4	10.9	8.5	Regional administrative systems

For peer review only

Hypertension, prior to stroke (%)	3.9	23.0	41.1	54.1	46.8	Regional administrative systems
Reperfusion treatment (thrombolysis or thrombectomy, %)	15.3	14.3	13.0	9.9	11.2	Swedish Stroke Register
Single household (%)	27.3	21.7	30.8	34.4	32.2	Statistics Sweden
Disposable income (EUR, nominal amount)						Statistics Sweden
Median	17 217	24 468	21 646	20 935	21 311	
IQR	12 542	15 470	15 386	14 983	15 062	
Educational level (%)						Statistics Sweden
≤9 years	16.95	13.79	27.37	31.04	28.27	
10-12 years	42.37	53.31	50	46.41	47.81	
≥12 years	40.68	32.9	22.63	22.55	23.92	
Born outside the EU (%)	10.0	15.0	15.6	8.7	11.1	Statistics Sweden
Sick leave the year prior to stroke (net days)	17.7	19.1	18.4	17.3	17.7	Swedish Social Insurance Agency

For peer review only

Disability pension the year prior to stroke (net days)	25.0	34.5	74.0	108.7	91.2	Swedish Social Insurance Agency
Total work absence the year prior to stroke (net days)	42.7 (StdErr 8.1)	53.6 (StdErr 4.9)	92.4 (StdErr 3.5)	126.0 (StdErr 2.1)		Swedish Social Insurance Agency

Note: “ADL dependent” stands for dependency regarding Activities of Daily Living, i.e. need for support to manage day-to-day activities including hygiene and mobility. Level of consciousness at hospital arrival is subject to judgement by responsible clinician. Disposable income is equivalent to total income minus taxes. IQR denotes the interquartile range and entails the difference between quartile 3 and quartile 1 for each of the age groups. The categories of educational level stand for the Swedish equivalents of high school, secondary school and post-secondary education.

For peer review only

For peer review only

Calculation of work absence and indirect costs

The individual levels of absence from work due to sickness the year prior to stroke varied between the age groups (Figure 1; confidence intervals available in Table A1). The youngest age groups showed average absence of 42.7 and 53.6 net days respectively, whilst individuals in ages 45-54 at stroke presented with 92.4 net days of absence the year prior to stroke. Individuals aged 55-63 at stroke had 126.0 net days of absence the year prior to stroke. The higher the age group, the higher the ratio of disability pension; the levels of net days prior to stroke were made up of 59% disability pension (<35 years), 64% disability pension (35-44 years), 80% disability pension (45-54 years) and 86% disability pension (55-63 years) respectively (the remaining proportion of net days regarded sickness benefit).

During the first year after stroke, levels of absence increased significantly from the year before, but overall, levels were on more similar levels across the different age groups; 153.1 (<35 years at stroke), 175.8 (35-44 years at stroke), 194.8 (45-54 years at stroke), and 211.8 (55-63 years at stroke) net days respectively. The increase between the year prior to stroke versus after stroke was highest in the youngest age group (3.6 times higher) and lowest in the eldest age group (1.7 times higher).

Levels of absence the second year after stroke were significantly lower than levels the first year after stroke. The largest relative drop in absence was found in the second youngest age group (35-44 years) and amounted to -33.5%. The age group with the lowest relative increase in the second year compared to the year prior to stroke was the eldest age group with 41% more net days.

Figure 1. Average health insurance levels utilized per individual, before and after stroke per age group for ischemic stroke (IS): net days of sick leave and/or disability pension. The y axis denotes number of net days of work absence, and the x axis denotes age groups expressed in years.

For peer review only

The productivity loss amounted to 110 Million Euros during the first year after stroke (Figure 2). The indirect costs were 27% lower during the second year after stroke and amounted to 76 Million Euros. The age-group shares of total were similar during the first and second year although the second youngest age group was relatively less costly during the second year after stroke compared to the first year.

The average indirect cost per participant per year during the first and second year amounted to 11.6 and 7.3 thousand Euros (kEUR) for the youngest age group, 16.6 kEUR and 9.0 kEUR for participants aged 35-44, 16.8 kEUR and 11.5 kEUR for participants aged 45-54, and 18.0 kEUR and 13.2 kEUR respectively for participants aged 55-63 at the time of stroke. On average, indirect monetary costs amounted to 17.4 kEUR per stroke case first year and 12.2 kEUR second year.

Figure 2. Indirect costs in terms of productivity loss per age group years 1-2 after stroke, based on individual income data from Statistics Sweden. Proportion based on population contribution reflects whether the age-group share of total costs is representative or not (age group under-represented if <100% and over-represented if >100%).

Statistical analysis of factors associated to work absence

When adjusting for clinical and sociodemographic factors potentially associated with levels of work absence after stroke, shown in Table 2, belonging to a certain age group was not significantly associated to differences in absence from work due to sick leave or disability pension.

Women had significantly fewer net days of absence during the year after stroke compared to men (p=0.040), after multivariable adjustments. Participants in the lowest (first) income quartile had significantly lower absence levels compared to participants in the second (p=0.000), third (p=0.000) and fourth (p=0.000) quartile. There were no significant differences for participants regarding educational level, region of birth or civil status. Performing the same regression analysis without previous health insurance utilization (net days of work absence the year prior to stroke) as a covariate implied significant differences, with several sociodemographic covariates statistically significantly associated to net days of work absence after stroke (Table A2).

For peer review only

Table 2. Association between patient characteristics and net days of absence (sick leave and/or disability pension). Coefficients and 95% CI from regression analysis.

	Coefficient	95% CI		p value
Sex, women	-0.085	-0.167	-0.004	0.040
Age				
<35	[reference]			
35-44	0.144	-0.114	0.402	0.274
45-54	0.158	-0.081	0.397	0.196
55-63	0.150	-0.085	0.386	0.211
ADL dependent	0.020	-0.299	0.340	0.902
Prior stroke (last two years)	-0.059	-0.257	0.140	0.561
Inpatient care the year prior to stroke	0.001	-0.002	0.004	0.562
Net days of absence the year prior to stroke (sick leave and/or disability pension)	0.003	0.003	0.003	0.000
Living situation				
At home	[reference]			
At home with home care	-0.067	-0.321	0.188	0.607
At special housing	-0.067	-0.436	0.303	0.724
Level of consciousness				
Conscious	[reference]			
Indolent	0.398	0.211	0.586	0.000
Unconscious	0.353	-0.083	0.790	0.113
Single household	0.013	-0.084	0.109	0.794

Disposable income				
Quartile 1	[reference]			
Quartile 2	0.309	0.201	0.417	0.000
Quartile 3	0.417	0.302	0.532	0.000
Quartile 4	0.218	0.097	0.340	0.000
Educational level				
≤9 years	[reference]			
10-12 years	0.022	-0.068	0.112	0.632
≥12 years	-0.079	-0.190	0.031	0.161
Born outside of the EU	0.033	-0.092	0.157	0.609
Marital status				
Married	[reference]			
Never married	0.076	-0.028	0.180	0.153
Divorced	0.030	-0.075	0.134	0.575
Widowed	-0.106	-0.366	0.154	0.425

Discussion

The burden associated with stroke is immense – in terms of Health-Related Quality of Life [1] as well as money [13-15]. In addition to severe personal suffering, stroke affects society by putting a significant burden on direct healthcare resources but also by indirect, major and long-lasting productivity loss. The first-year indirect monetary costs for stroke in the present study totaled 110 Million Euros. Given that almost two thirds of the Swedish stroke cases were the basis for analysis and that both urban and rural areas are represented, it is likely not unfair to make an extrapolation for all of Sweden; assuming equivalent incidence and equivalent levels of income for all of Sweden renders an estimate of approximately 169 Million Euros annually – including only the first year after stroke. The costs are not limited to the first or second year (indirect monetary costs of 76 MEUR for the study population, estimated to 117 MEUR for the full Swedish population), but expected to last several years after the stroke event [16]. Information on monetary productivity loss per year due to stroke in other countries is scarce, as shown in previous literature review [17], but significantly lower levels than what was found in this study has been reported (e.g. £ 2 175 per case for UK [18]). A study from Denmark, a context similar to the present study, estimated the indirect costs to 3.1 kEUR, although the majority of the subjects was no longer working (58.2% above 70 years of age) [19]. Indirect costs related to stroke incidence in Sweden have been assessed in previous studies and found to be a minor proportion of the total cost, amounting to 1.99-5.94 kEUR per stroke first year [13-15]. These studies did however include persons of all ages afflicted with stroke, and likely the indirect costs are significantly higher for the group in general working age as included in the present study. The estimate of total indirect costs of stroke in Sweden in this study is on par with a 2020 published estimate of 122 MEUR (productivity loss due to morbidity) [20].

Levels of sick leave the year prior to stroke were similar between the age groups (17.3-19.1 net days), whilst absence due to disability pension the year prior to stroke varied significantly (25.0-108.7 net days); the older the age group, the higher absolute and relative levels of disability pension. The levels of net absence during the year prior to stroke points at the fact that participants active on the labor market were more afflicted with disease than the average general population; pre-stroke levels of sick leave (absence excluding disability pension) in the study population amounted to 17.7 net days (average of all age groups) whilst the national average for the same time period amounted to 6.0 net days (2010) [21]. The patterns are the same looking at the health insurance as a whole; total absence in the study population the year prior to stroke amounted to more than three times the total absence in the general population for the study population (109.0 net days compared to 29.5 net days 2010 [21]). The proportion of women over the different age groups differed; the younger the age group, the higher the proportion of women. The reasons for this are several; hypertension and atrial fibrillation are less frequent in men than in women, whilst risk factors such as atrial fibrillation are of higher impact in women. In addition, pregnancy and contraceptives affect hormone levels and increase the risk for stroke [22].

Sex and level of consciousness at hospital arrival were significantly associated with the level of absence the year after stroke; women were less absent on sick leave and/or disability pension as were participants who were conscious at hospital arrival compared to those who were indolent. The finding that female sex was significantly associated with lower levels of absence the year after stroke stood out compared to the general population; according to the Social Insurance Agency, women have higher risk for being on sick leave, a difference which has continued to increase since 2010 [23]. Sick leave figures in public reports are often presented without multivariable adjustments which was the case in the present study. Furthermore, disposable income showed strong association with

For peer review only - http://bmjopen.bmj.com/site/about/guidelines.xhtml

absence after stroke; participants in the lowest income group had significantly lower levels of absence compared to all other income groups.

Absence the year prior to stroke was positively correlated with absence the year after stroke. Interestingly, when this baseline characteristic was not taken into account, several socioeconomic indicators showed significant association with absence the year after stroke, including educational level, birth region and civil status.

There are several limitations with this study. Informal care is costly, and no estimation of informal care has been included in this study due to lack of reliable data. Furthermore, the actual costs measured are also in fact higher as any cases of sick benefits lasting shorter than 14 days are not registered in the Social Insurance Agency database. There is also a lack of information whether the individuals were employed at the time of stroke or not, which would have been valuable to nuance the perspective on health insurance utilization versus unemployment. The data may also be regarded as old as the first data point is more than 10 years old at the time of publication.

There are also several strengths with the present study. The underlying data are to a large extent extracted from population registries which implies very high coverage rates and hence a close-to-true picture of the actual population, and sick-leave costs for stroke patients have been calculated before as well as after the stroke event, which adds important information in terms of incremental costs after stroke. The possibility to adjust for several factors such as sex, age, comorbidity as well as several sociodemographic factors enables a better understanding of what patient characteristics that are in fact associated with health insurance utilization. The computation of productivity loss is based on objective data points for registered work absence and not related to any preference-based measures for health measures. Therefore, the findings regarding indirect costs related to stroke from the present study could be used together with preference-based measures and estimations of impact on health-related quality of life from other studies without implying risk for double counting, in line with previously published recommendations [24].

During 2008 significant changes were implemented in the Swedish health insurance to limit the length of time possible to receive support for loss of income due to disability. These changes were implemented at the beginning of the time period assessed in the present study and could be expected to influence the estimation of costs equivalently over the time period studied [25].

The fact that low-income individuals had fewer net days of sick leave is important to note as this points to a socioeconomic gradient in the utilization of the general health insurance. As health equity is a topic treated in the Swedish healthcare act it is particularly relevant to evaluate whether the distribution of governmental funding is actually equitable. Disposable income is an example of a factor that should not be associated to social insurance utilization after consideration has been taken to medical factors. There are several different factors that may impact and drive that gradient, but it is likely that factors such as higher general dependence on income in lower-income professions and higher general knowledge regarding the social insurance system in higher-income groups are of relevance to explain this gradient [26]. It is also likely that underlying themes such as knowledge of and ability to pursue primary and secondary stroke prevention is asymmetrically distributed across e.g. income groups. Such patterns reenforces already existing systematic differences in ill-health, and actions to impact that are key to change the situation.

Conclusions

For peer review only

Individuals that are afflicted with stroke in working age have a significantly higher pre-stroke level of health insurance (sick leave or disability pension) utilization than the average in Sweden. The monetary burden of stroke is very high; the first-year indirect costs for an individual in working age amounts to more than one third of the GDP per capita (reference year 2011). There is possibly a socioeconomic gradient in the utilization of Swedish health insurance among stroke patients; high-income individuals generally had higher levels of sick leave than did low-income individuals, while simultaneously adjusting for other relevant factors on patient level. Continuous assessments of the social insurance utilization among stroke patients – as well as for other conditions – by governmental institutions are encouraged, to follow up on the equity in its design and practical implications.

For peer review only

List of abbreviations

ADL – activities of daily living

CI – confidence interval

EU – European Union

EUR – Euros

GDP – Gross Domestic Product

ICD-10 - International Classification of Diseases, 10th revision

IQR – interquartile range

IS – ischemic stroke

kEUR – thousand Euros

MEUR – Million Euros

mRS – modified Rankin Scale

NIHSS – National Institutes of Health Stroke Severity

Contributorship

CW and KSS conceived the idea to the study. CW performed the analyses and drafted the manuscript. CW, EW, FB, MvE and KSS contributed to the final version and approved the submitted version.

Competing interests

None declared.

Funding

This study was supported by grants from the Swedish Research Council (VR2017-00946), the Swedish Heart and Lung Foundation, the Swedish Brain Foundation, the Promobilia Foundation, the

Norrbacka-Eugenia Foundation as well as by grants from the Swedish state under an agreement between the Swedish government and the county councils, the ALF agreement (ALFGBG-718711).

Data sharing

The dataset generated and analysed during the current study is not publicly available due to Swedish law; access to the dataset was given to the researchers under the condition of timely valid ethical approval. Additional information regarding the availability is available from the corresponding author upon request.

Acknowledgements

We are grateful to the Swedish Stroke Register (www.riksstroke.org) and the different healthcare regions for providing data for this study. We also wish to thank all patients, caregivers, reporting units, and coordinators in Riksstroke as well as Riksstroke steering committee.

Patient and Public Invlovement

It was not appropriate or possible to involve patients or the public in the design, or conduct, or reporting, or dissemination plans of our research.

For peer review only

References

1. *Global, regional, and national burden of stroke, 1990-2016: a systematic analysis for the Global Burden of Disease Study 2016.* Lancet Neurol, 2019. **18**(5): p. 439-458 GBD Stroke Collaborators.
2. Luengo-Fernandez, R., et al., *Economic burden of stroke across Europe: A population-based cost analysis.* European Stroke Journal, 2019: p. 2396987319883160.
3. Ervasti, J., et al., *Permanent work disability before and after ischaemic heart disease or stroke event: a nationwide population-based cohort study in Sweden.* BMJ Open, 2017. **7**(9): p. e017910.
4. *The Swedish social insurance during 2018 (Socialförsäkringen i siffror 2018)*. 2018, The Swedish Social Insurance Agency: Stockholm.
5. *About the Swedish social insurance*, T.S.S.I. Agency, Editor. 2019.
6. *Average age of retirement and exit from the Swedish labor market (Medelpensioneringsålder och utträdesålder)*. 2016, The Swedish Pensions Agency.
7. Söderholm, A., et al., *Validation of Hospital Performance Measures of Acute Stroke Care Quality. Riksstroke, the Swedish Stroke Register.* Neuroepidemiology, 2016. **46**(4): p. 229-34.
8. Eriksson, M., et al., *Assessment of functional outcome in a national quality register for acute stroke: can simple self-reported items be transformed into the modified Rankin Scale?* Stroke, 2007. **38**(4): p. 1384-6.
9. Bray, B.D., et al., *Derivation and external validation of a case mix model for the standardized reporting of 30-day stroke mortality rates.* Stroke, 2014. **45**(11): p. 3374-80.
10. Drummond, M.F., *Methods for the economic evaluation of health care programmes*. 3rd edition ed. 2005, Oxford: Oxford University Press.
11. *Size of employer contributions*. [cited 2019 25 October]; Description of employer contribution for persons in general working age and its components:[Available from: https://www4.skatteverket.se/rattsligvagledning/edition/2014.14/1333.html.
12. *Oanda Currency Converter*. 2019 [cited 2019 November 14]; Available from: https://www1.oanda.com/currency/converter/.
13. Lekander, I., et al., *Relationship between functional disability and costs one and two years post stroke.* PLoS One, 2017. **12**(4): p. e0174861.
14. Ghatnekar, O., et al., *Cost of stroke in Sweden: an incidence estimate.* Int J Technol Assess Health Care, 2004. **20**(3): p. 375-80.
15. Ghatnekar, O., et al., *Costs for stroke in Sweden 2009 and developments since 1997.* Int J Technol Assess Health Care, 2014. **30**(2): p. 203-9.
16. Gloede, T.D., et al., *Long-term costs of stroke using 10-year longitudinal data from the North East Melbourne Stroke Incidence Study.* Stroke, 2014. **45**(11): p. 3389-94.
17. Luengo-Fernandez, R., A.M. Gray, and P.M. Rothwell, *Costs of stroke using patient-level data: a critical review of the literature.* Stroke, 2009. **40**(2): p. e18-23.
18. Saka, O., A. McGuire, and C. Wolfe, *Cost of stroke in the United Kingdom.* Age Ageing, 2009. **38**(1): p. 27-32.
19. Jennum, P., et al., *Cost of stroke: a controlled national study evaluating societal effects on patients and their partners.* BMC Health Serv Res, 2015. **15**: p. 466.
20. Luengo-Fernandez, R., et al., *Economic burden of stroke across Europe: A population-based cost analysis.* Eur Stroke J, 2020. **5**(1): p. 17-25.
21. *Social Insurance Report 2011:6*. 2011, The Swedish Social Insurance Agency.
22. Cordonnier, C., et al., *Stroke in women - from evidence to inequalities.* Nat Rev Neurol, 2017. **13**(9): p. 521-532.
23. *Social Insurance Report 2018:2*. 2018, The Swedish Social Insurance Agency.
24. Sanders, G.D., et al., *Recommendations for Conduct, Methodological Practices, and Reporting of Cost-effectiveness Analyses: Second Panel on Cost-Effectiveness in Health and Medicine.* Jama, 2016. **316**(10): p. 1093-103.

For peer review only

25. *Follow-up report of the health insurance reform, reference number 037795-2011 (Försäkringskassans uppföljning av sjukförsäkringsreformen – delredovisning)*. 2011, The Swedish Social Insurance Agency.

26. *Insured but not reimbursed - a report on the under-utilization of the union-based health insurance (Försäkrad men utan ersättning - en granskning av underutnyttjandet av den kollektivavtalade sjukförsäkringen); 2018:7*. 2018, The Swedish Social Insurance Inspectorate.

For peer review only

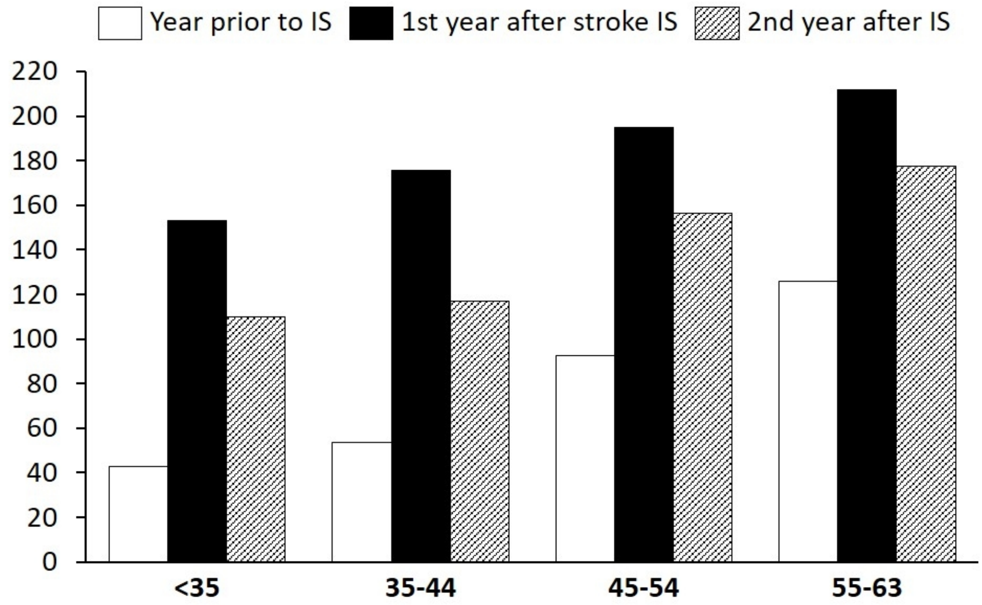

Figure 1. Average health insurance levels utilized per individual, before and after stroke per age group for ischemic stroke (IS): net days of sick leave and/or disability pension. The y axis denotes number of net days of work absence, and the x axis denotes age groups expressed in years.

80x51mm (300 x 300 DPI)

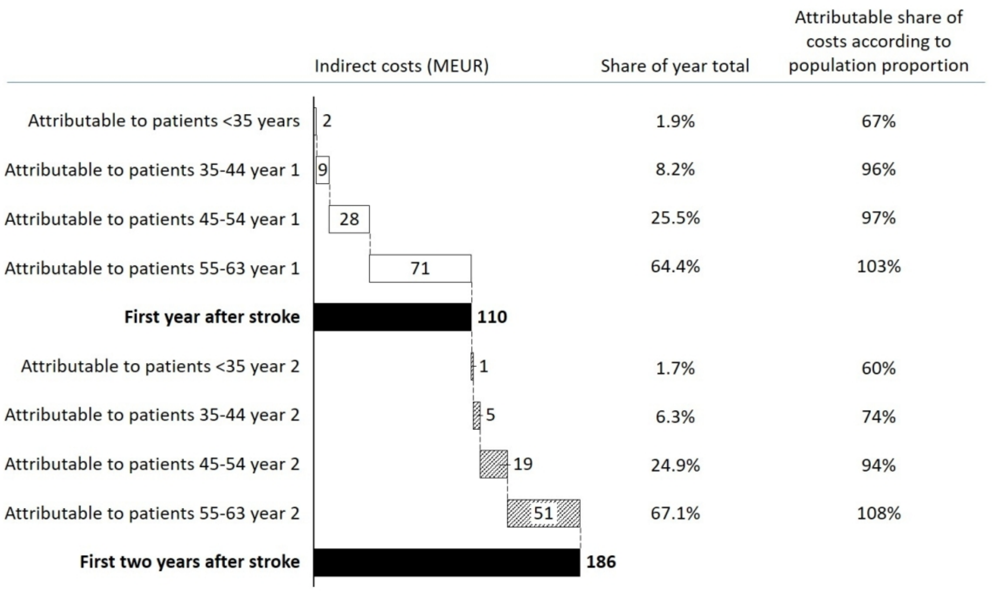

Figure 2. Indirect costs in terms of productivity loss per age group years 1-2 after stroke, based on individual income data from Statistics Sweden. Proportion based on population contribution reflects whether the age-group share of total costs is representative or not (age group under-represented if <100% and over-represented if >100%).

123x74mm (300 x 300 DPI)

Table A1. Means and 95% confidence intervals for levels of work absence during the year prior to stroke and the two years following a stroke.

	**Mean**	**95% CI**	
Year prior to ischemic stroke			
<35	42.68409	26.76095	58.60723
35-44	53.65557	44.05273	63.25841
45-54	92.40803	85.53612	99.27993
55-63	125.9772	121.0704	130.8841
First year after ischemic stroke			
<35	153.0585	131.2016	174.9154
35-44	175.7974	163.4365	188.1584
45-54	194.7995	187.5116	202.0874
55-63	211.7774	206.9247	216.6301
Second year after ischemic stroke			
<35	109.7589	86.66959	132.8482
35-44	116.8577	104.0181	129.6973
45-54	156.4038	148.4572	164.3505
55-63	177.6091	172.3538	182.8644

Table A2. Sensitivity analysis of multivariable regression assessing association between patient characteristics and net days of absence (sick leave and/or disability pension). Coefficients and 95% CI from regression analysis.

	**Coefficient**	**95% CI**		**p value**
Sex, women	-0,16113	-0,24734	-0,07493	0.000
Age				
<35	[reference]			
35-44	0,188983	-0,10058	0,478549	0,201
45-54	0,250684	-0,01593	0,517297	0,065
55-63	0,175926	-0,08461	0,436462	0,186
ADL dependent	0,12426	-0,19632	0,444836	0,447
Prior stroke (last two years)	0,166235	-0,0371	0,369573	0,109
Inpatient care the year prior to stroke	0,004202	0,00082	0,007583	0,015
Living situation				
At home	[reference]			
At home with home care	0,157154	-0,09757	0,411879	0,227
At special housing	0,186942	-0,19707	0,570954	0,34
Level of consciousness				
Conscious	[reference]			
Indolent	0,322118	0,122908	0,521328	0,002
Unconscious	0,331179	-0,1402	0,802554	0,169
Single household	0,037734	-0,06697	0,14244	0,48
Disposable income				

Quartile 1	[reference]			
Quartile 2	-0,03551	-0,15224	0,08123	0,551
Quartile 3	0,320181	0,18418	0,456183	0,000
Quartile 4	0,235572	0,125605	0,34554	0,000
Educational level				
≤9 years	[reference]			
10-12 years	-0,04666	-0,14196	0,048639	0,337
≥12 years	-0,23271	-0,34721	-0,1182	0,000
Born outside of the EU	0,161965	0,022217	0,301714	0,023
Marital status				
Married	[reference]			
Never married	0,15266	0,03777	0,26755	0,009
Divorced	0,113267	0,001084	0,225449	0,048
Widowed	-0,15581	-0,40183	0,090218	0,215

STROBE Statement—checklist of items that should be included in reports of observational studies

	Item No.	Recommendation	Page No.
**Title and abstract**	1	(*a*) Indicate the study's design with a commonly used term in the title or the abstract	1
		(*b*) Provide in the abstract an informative and balanced summary of what was done and what was found	2
**Introduction**			
Background/rationale	2	Explain the scientific background and rationale for the investigation being reported	3
Objectives	3	State specific objectives, including any prespecified hypotheses	3
**Methods**			
Study design	4	Present key elements of study design early in the paper	3
Setting	5	Describe the setting, locations, and relevant dates, including periods of recruitment, exposure, follow-up, and data collection	3
Participants	6	(*a*) *Cohort study*—Give the eligibility criteria, and the sources and methods of selection of participants. Describe methods of follow-up<br>*Case-control study*—Give the eligibility criteria, and the sources and methods of case ascertainment and control selection. Give the rationale for the choice of cases and controls<br>*Cross-sectional study*—Give the eligibility criteria, and the sources and methods of selection of participants	3-5
		(*b*) *Cohort study*—For matched studies, give matching criteria and number of exposed and unexposed<br>*Case-control study*—For matched studies, give matching criteria and the number of controls per case	
Variables	7	Clearly define all outcomes, exposures, predictors, potential confounders, and effect modifiers. Give diagnostic criteria, if applicable	4
Data sources/ measurement	8*	For each variable of interest, give sources of data and details of methods of assessment (measurement). Describe comparability of assessment methods if there is more than one group	3-4
Bias	9	Describe any efforts to address potential sources of bias	4-5
Study size	10	Explain how the study size was arrived at	3

Continued on next page

For peer review only - http://bmjopen.bmj.com/site/about/guidelines.xhtml

Quantitative variables	11	Explain how quantitative variables were handled in the analyses. If applicable, describe which groupings were chosen and why	4-5
Statistical methods	12	(*a*) Describe all statistical methods, including those used to control for confounding	4-5
		(*b*) Describe any methods used to examine subgroups and interactions	4-5
		(*c*) Explain how missing data were addressed	5
		(*d*) *Cohort study*—If applicable, explain how loss to follow-up was addressed<br>*Case-control study*—If applicable, explain how matching of cases and controls was addressed<br>*Cross-sectional study*—If applicable, describe analytical methods taking account of sampling strategy	
		(*e*) Describe any sensitivity analyses	
Participants	13*	(a) Report numbers of individuals at each stage of study—eg numbers potentially eligible, examined for eligibility, confirmed eligible, included in the study, completing follow-up, and analysed	3, 7
		(b) Give reasons for non-participation at each stage	3, 5
		(c) Consider use of a flow diagram	
Descriptive data	14*	(a) Give characteristics of study participants (eg demographic, clinical, social) and information on exposures and potential confounders	7-8
		(b) Indicate number of participants with missing data for each variable of interest	7
		(c) *Cohort study*—Summarise follow-up time (eg, average and total amount)	3
Outcome data	15*	*Cohort study*—Report numbers of outcome events or summary measures over time	7-10
		Case-control study—Report numbers in each exposure category, or summary measures of exposure	
		Cross-sectional study—Report numbers of outcome events or summary measures	
Main results	16	(*a*) Give unadjusted estimates and, if applicable, confounder-adjusted estimates and their precision (eg, 95% confidence interval). Make clear which confounders were adjusted for and why they were included	7-10
		(*b*) Report category boundaries when continuous variables were categorized	7
		(*c*) If relevant, consider translating estimates of relative risk into absolute risk for a meaningful time period	

Continued on next page

For peer review only

Other analyses	17	Report other analyses done—eg analyses of subgroups and interactions, and sensitivity analyses	10
Key results	18	Summarise key results with reference to study objectives	9-10
Limitations	19	Discuss limitations of the study, taking into account sources of potential bias or imprecision. Discuss both direction and magnitude of any potential bias	14
Interpretation	20	Give a cautious overall interpretation of results considering objectives, limitations, multiplicity of analyses, results from similar studies, and other relevant evidence	13-14
Generalisability	21	Discuss the generalisability (external validity) of the study results	13
**Other information**			
Funding	22	Give the source of funding and the role of the funders for the present study and, if applicable, for the original study on which the present article is based	15

*Give information separately for cases and controls in case-control studies and, if applicable, for exposed and unexposed groups in cohort and cross-sectional studies.

Note: An Explanation and Elaboration article discusses each checklist item and gives methodological background and published examples of transparent reporting. The STROBE checklist is best used in conjunction with this article (freely available on the Web sites of PLoS Medicine at http://www.plosmedicine.org/, Annals of Internal Medicine at http://www.annals.org/, and Epidemiology at http://www.epidem.com/). Information on the STROBE Initiative is available at www.strobe-statement.org.

For peer review only
